# A Language Performance Model for Predicting Glioma Recurrence and Molecular Biomarkers: A Retrospective Cohort Study

**DOI:** 10.1002/brb3.71243

**Published:** 2026-03-02

**Authors:** Hua Song, Linghao Bu, Chen Luo, Luhao Yang, Shuai Wu, Jie Zhang, Ye Yao

**Affiliations:** ^1^ Department of Biostatistics, School of Public Health Fudan University Shanghai China; ^2^ Department of Neurosurgery, The First Affiliated Hospital Zhejiang University School of Medicine Hangzhou China; ^3^ Department of Neurosurgery, Huashan Hospital, Shanghai Medical College Fudan University Shanghai China

**Keywords:** glioma, pathology, molecular, language disorders, survival analysis

## Abstract

**Purpose:**

Glioma progression is often accompanied by language dysfunction, and postoperative recurrence is nearly inevitable, especially in high‐grade cases. This study aimed to identify valuable language‐based prognostic markers and improve risk management strategies.

**Methods:**

A retrospective longitudinal cohort of 191 glioma patients (2010–2018) was analyzed. Language status was assessed using the Aphasia Battery of Chinese (ABC), adapted from the Western Aphasia Battery. Principal component analysis (PCA) addressed collinearity in language scores, and Cox regression identified predictors for a survival model. Bootstrap validation and SHapley Additive exPlanations (SHAP) were used to confirm model stability and interpretability. Logistic regression was used to test associations between predictors and pathological molecular parameters.

**Results:**

Auditory verbal comprehension & writing and repetition emerged as key language predictors in the Cox model, achieving an AUC of 0.834. SHAP analysis confirmed their dominant contribution, defining the model as the Language Tests Combinations (LTC) model. Two‐sample *t*‐tests revealed significant differences in language scores between IDH1/2‐mutant and wild‐type groups. Logistic regression showed strong correlations between language predictors and molecular parameters (MGMT codeletion: AUC = 0.922; 1p/19q: AUC = 0.813; IDH1/2: AUC = 0.748), with tumor locations included as dummy variables.

**Conclusion:**

Language components were identified as robust predictors of glioma recurrence and were significantly associated with key molecular features. The LTC model represents an internally validated and interpretable exploratory prognostic framework that may complement existing risk‐stratification approaches and inform future studies on postoperative glioma management.

## Graphical abstract

This study proposes a language‐based prognostic model for glioma recurrence by analyzing preoperative language performance using the Aphasia Battery of Chinese in 191 patients with WHO grade 2–4 gliomas. Principal component analysis and Cox regression identified key language predictors, which were used to construct and internally validate the language tests combinations (LTC) model. The model demonstrated strong predictive power and significant associations with molecular biomarkers, offering a noninvasive tool for risk stratification and personalized management.

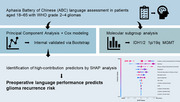

**Abbreviations**
1p/19q‐codeletion: complete deletion of both the short arm of chromosome 1 (1p) and the long arm of chromosome 19 (19q)
ABC: Aphasia Battery of Chinese
AQ: Aphasia Quotient
AUC: area under the receiver operating characteristic curve
CPH: Cox proportional‐hazards
CQ: Cortical Quotient
IDH1/2: isocitrate dehydrogenase 1/2
KPS: Karnofsky performance scale
LTC: language tests combination
MGMT: O‐6‐methylguanine‐DNA methyltransferase
MMSE: mini‐mental state examination
PFS: progression‐free survival
SHAP: SHapley Additive exPlanations.
WAB: Western Aphasia Battery

## 1 Introduction

Gliomas are the most prevalent primary malignant brain tumors in adults, accounting for approximately 81% of these malignancies (Kudulaiti et al. 2021). Despite optimal treatment, the median progression‐free survival for patients with glioblastoma is less than 12 months (Vaz‐Salgado et al. 2023). Postoperative recurrence is nearly inevitable, especially for higher‐grade gliomas. Previous studies have found that the progression of gliomas is associated with a loss of cognitive functions (Kelly, Majewska, Ioannidis, Raza, and Williams 2017; Vaz‐Salgado et al. 2023). This high mortality rate and inherently disabling effect on patients have imposed a high burden of disease and economics on society and families (Abbafati et al. 2020; Ohgaki and Kleihues n.d.; Ostrom et al. 2014; Patel et al. 2019). Therefore, it is crucial to develop individualized treatment strategies for patients with varying recurrence risks, and the choice of surgery, radiotherapy, or conservative treatment should be determined as early as possible. Achieving this goal may involve identifying accessible, cost‐effective, and reliable preoperative predictors, which can then be incorporated into predictive models.

Existing research on predictors of glioma recurrence has primarily focused on factors such as age, sex, Karnofsky performance status (KPS), and tumor grade (including histomolecular classification) (Lacroix et al. 2001; Thakkar et al. 2014; van Kessel et al. 2021). However, prediction models using multimodal neuroimaging were affected by technical parameters and equipment variations, necessitating extensive standardization and calibration efforts (Liu and Liu 2023). Recent studies have demonstrated the potential of high‐dimensional imaging features for prognostic prediction. Specifically, MRI radiomics was used to predict 1p/19q co‐deletion status in low‐grade glioma, and transfer learning on MRI radiomics signatures was applied to predict overall survival across glioma subtypes, highlighting how advanced imaging‐based approaches can noninvasively capture molecular and prognostic information (Kha et al. 2021; Le Minh, Kha, and Le 2023). Consequently, better predictors of prognosis are needed for personalized therapeutic decision‐making. Recent findings indicate that language function may serve as a marker of glioma characteristics and prognosis. This is not surprising given that the frequent locations of gliomas involve language eloquent areas, resulting in aphasia and other neurological deficits (Duffau and Capelle 2004; Gómez Vecchio et al. 2021). Previous studies also demonstrate that the severity of language impairment is susceptible to tumor grade(Yuan et al. 2023, Yuan et al. 2020), as well as molecular profiles (Soldatelli et al. 2022; van Kessel et al. 2022; K. Zhang et al. 2024). For example, IDH‐wildtype gliomas are more likely to cause language deficits compared with IDH‐mutant tumors (Wefel et al. 2016). Language assessments are noninvasive, convenient, and not dependent on specialized equipment. To date, there is no study using language functional status as a predictor of glioma prognosis, despite the fact that there are readily available language function measures, such as AQ (Aphasia Quotient) and CQ (Cortical Quotient), in terms of language function tests. This gap highlights the need for further research into using language function as a reliable prognostic predictor.

Artificial intelligence (AI), including machine learning (ML) and deep learning (DL) algorithms, has recently emerged as a transformative force in neurosurgery and neuro‐oncology, demonstrating considerable potential in tumor segmentation, non‐invasive molecular characterization, survival and recurrence prediction, and personalized treatment planning for gliomas (Mohammadzadeh et al. 2025; Mohammadzadeh, Niroomand, et al. 2025). Increasing evidence indicates that AI‐based models integrating radiological, clinical, and molecular data can reliably predict key biomarkers such as IDH mutation and MGMT promoter methylation, thereby significantly improving diagnostic and classification accuracy without the need for invasive sampling (Alhasan n.d.; Evangelou et al. 2025; Tbahriti et al. 2025). In clinical management, these AI‐driven advances may shorten the interval from diagnosis to treatment initiation, enhance preoperative risk stratification, and assist surgical decision‐making, highlighting their growing relevance in modern neuro‐oncologic practice.

Given this background, we conducted an exploratory analysis using easily obtainable functional data based on traditional statistical modeling. Positive findings from such approaches may, in the future, provide supportive evidence or complementary perspectives for AI‐based imaging or “omics”‐driven models, particularly in clinical settings where advanced neuroimaging or molecular testing is not immediately accessible. Preoperative prognostic prediction provides patients with clearer expectations and enables them to participate more actively in treatment decisions. A more accurate understanding of prognosis allows patients and their families to plan comprehensively at an early stage rather than responding passively to postoperative recurrence risks or functional decline. In this study, we hypothesize that specific patterns of language dysfunction can predict glioma recurrence, survival, and molecular characteristics. We analyzed preoperative behavioral performance together with postoperative recurrence outcomes and identified functional predictors most closely associated with prognosis. These predictors were subsequently used to construct a survival prediction model. In addition, we proposed an approach for preoperatively inferring three clinically important molecular parameters (IDH1/2 mutation, MGMT promoter methylation, and 1p/19q codeletion) based on patients’ language performance. Our findings highlight the prognostic value of language function and offer a novel perspective for risk assessment, supporting more personalized rehabilitation planning. Patients with a more favorable prognosis may safely follow standard treatment and recovery pathways, whereas high‐risk patients could benefit from enhanced preoperative intervention and rehabilitation strategies aimed at delaying disease progression and improving quality of life.

## 2 Materials and Methods

### 2.1 Participants and Study Design

**FIGURE 1:**
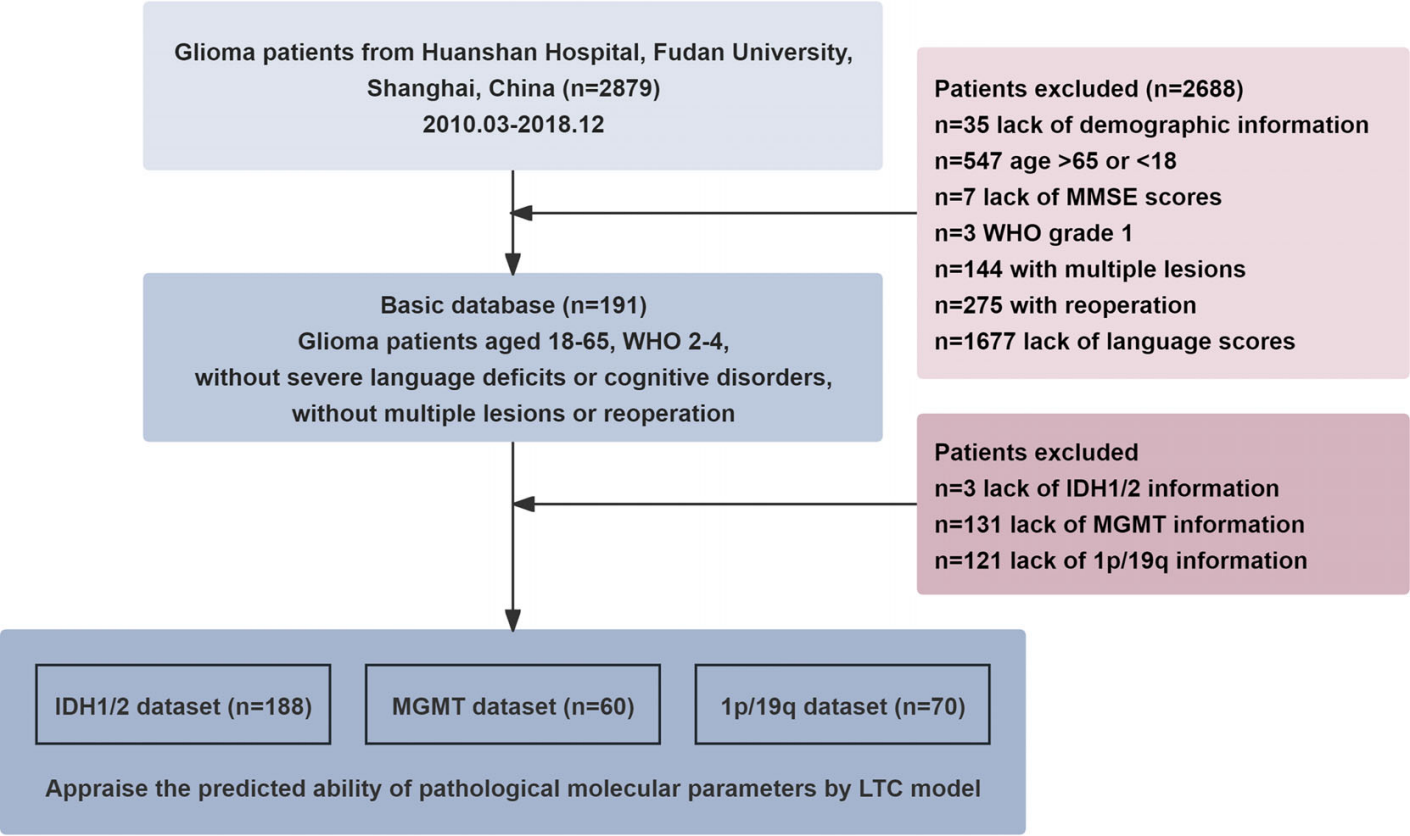
Flowchart generated for inclusion and exclusion criteria processing.

Patients were recruited from the Glioma Surgery Division in the Neurological Surgery Department at Huashan Hospital, Shanghai Medical College, Fudan University, Shanghai, China. From March 2010 to December 2018, 191 patients with detailed demographic and behavioral data were included. The inclusion criteria were as follows: native Chinese speakers aged 18 to 65 years, right‐handed, diagnosed with diffuse glioma of WHO 2–4 (2021 WHO version) in the left frontal, temporal, parietal, occipital, or insular lobe. Patients with multiple lesions, repeated surgeries, severe language deficits (AQ<50), or cognitive disorders (mini‐mental state examination [MMSE]<14) were excluded. A detailed flowchart outlining the exclusion process and inclusion criteria is presented in Figure 1.

Demographic information (i.e., age at diagnosis, sex, and education level), clinical data (i.e., lesion location, chief complaint, WHO grade, and molecular profile of glioma), and follow‐up data were retrieved from the electronic information system of Huashan Hospital from March 2010 to December 2018. A thorough follow‐up protocol starts with regular follow‐up after maximal safe tumor resection until death or loss of follow‐up. As the endpoint of this study, PFS was defined as the time from the date of tumor resection to the date of glioma recurrence, while recurrence was determined using the Response Assessment in Neuro‐Oncology (RANO) criteria (Nayak et al. 2017). Considering the 12‐month PFS rate of glioma patients was 74.2% in the existing evidence, many glioma patients, especially those with high‐grade glioma, faced an elevated risk of recurrence within the 12 months after surgery (Kong et al. 2020). Therefore, selecting 12 months as the prediction time point can effectively evaluate the short‐term effects of surgery and treatment (Vecchio et al. 2024). This relatively brief follow‐up period allows clinicians and researchers to monitor recurrence risk within a reasonable timeframe, offering a foundation for adjustments to patients’ ongoing treatment plans.

### 2.2 Language and Neuropsychological Testing

Behavioral scores of all patients were obtained for the day before the surgery. MMSE was utilized to evaluate the cognitive states of patients. Functional impairment was assessed by the Karnofsky performance scale (KPS) (Karnofsky et al. 1948). Language function was assessed using the Aphasia Battery of Chinese (ABC) (Gao et al. 1992). This instrument was developed with reference to the Western Aphasia Battery (WAB) and incorporates commonly used Chinese words and sentences, avoiding rare or syntactically complex items. Validation studies have shown no significant differences across sex, age, handedness, or educational groups in any of its subtests (Gao 1992). The ABC is therefore considered a standardized and appropriate aphasia assessment tool for Mandarin‐speaking populations (Huang et al. 2021; N. Zhang et al. 2018). Overall language functions were assessed with six tests, including “spontaneous speech”, “repetition”, “naming”, “auditory verbal comprehension”, “reading”, and “writing” (Table S1). The Cortical Quotient (CQ) is a weighted average of all subtest scores. The Aphasia Quotient (AQ) is a weighted average of all subtest scores relating to spoken language (spontaneous speech, naming, repetition, and auditory verbal comprehension). The scores have been normalized to percentages relative to the maximum attainable score for each test, ensuring uniformity and facilitating comprehension. All patients underwent assessments performed by an experienced neuropsychologist. The scoring criteria and description of tests are presented in Table S1.

### 2.3 Statistical Analyses

Continuous variables were shown as mean [standard deviation, SD] in patients with statistical significance based on a two‐sample *t*‐test, while categorical variables were shown as percentage with statistical significance based on the Chi‐squared test (χ ^2^ & *p*) and the Fisher exact test (z & *p*). Cox proportional hazards regression was applied to identify predictors associated with recurrence and to construct survival models. Hazard ratios (HRs) and corresponding 95% confidence intervals (CIs) were estimated, and the statistical significance of individual covariates was evaluated using the Wald test. Differences in survival curves between recurrence risk groups were assessed using the log‐rank test. For the language scores selected in the model, principal component analysis (PCA) was applied to reduce multicollinearity among the language‐related variables and to obtain a more parsimonious representation of language function. The number of principal components retained was determined by balancing information preservation and model stability, rather than relying on a rigid cumulative variance cutoff (Abdi and Williams 2010; Jolliffe 2002). This approach aimed to retain the majority of variance in the original language scores while limiting model complexity, particularly given the moderate sample size. Sensitivity analyses using different numbers of principal components were subsequently conducted to evaluate the prediction of model performance. The contribution of the predictors to the model classification was represented using SHapley Additive exPlanations (SHAP) analyses. Considering data protection constraints, this single‐center study utilized the Bootstrap method to validate the model internally. A corresponding nomogram was formulated to predict postoperative recurrence within 12 months. Logistic models were utilized to predict molecular parameters, with the language part of the predictors selected before. The sensitivity and specificity of the models were evaluated by ROC curves. The area under the receiver operating characteristic curve (AUC) was chosen as the measurement to compare the performances of models. The confidence interval of the AUC is calculated using the Bootstrap method. The ROC curve with the larger AUC is considered to have a better predicted performance (Nahm 2022). *p* < 0.05 was considered statistically significant. Statistical analyses were performed using Python version 3.6.3 and R version 4.2.3 software.

## 3 Results

### 3.1 Patient Characteristics

The 191 glioma patients included 113 (59.16%) male and 78 (40.84%) female, with a mean age of 42.75 [10.54] years. 40 (20.94%) experienced recurrence before the endpoint of the follow‐up period, and 22 (11.52%) relapsed within 12 months. There were no statistically significant differences in age at diagnosis, sex, or education level. Moreover, compared with the patients without recurrence, the recurrence group exhibited significantly lower scores in MMSE, AQ, CQ, spontaneous speech, repetition, naming, auditory verbal comprehension, and reading. Detailed characteristics of scores and corresponding statistical results were summarized in Table 1.

**TABLE 1:** Baseline characteristics of glioma patients overall and in the PFS and recurrence groups.

Demographic and clinical data	Overall (*n* = 191)	PFS/Recurrence			
		PFS (*n* = 151)	Recurrence (*n* = 40)	t/z/χ ^2^	*p*‐value
Age at diagnosis (years), mean ± SD	42.75 ± 10.54	42.26 ± 9.89	44.58 ± 12.69	−1.23	0.219
Sex male/female	113/78	86/65	27/13	1.46	0.228
Education (years), mean ± SD	12.40 ± 3.12	12.29 ± 2.89	12.80 ± 3.88	−0.77	0.443
WHO grade 2, *n* (%)	116 (60.73)	110 (72.85)	6 (15.00)	44.37	2.9 × 10^−^^1^ ^1^**
WHO grade 3, *n* (%)	30 (15.71)	18 (11.92)	12 (30.00)	7.81	0.005*
WHO grade 4, *n* (%)	45 (23.56)	23 (15.23)	22 (55.00)	27.77	1.9 × 10^−7^**
Location (frontal lobe), *n* (%)	89 (46.60)	78 (51.66)	11 (27.50)	7.42	0.006*
Location (temporal lobe), *n* (%)	27 (14.14)	17 (11.26)	10 (25.00)	4.92	0.027*
Location (parietal lobe), *n* (%)	15 (7.85)	9 (5.96)	6 (15.00)	2.43	0.119
Location (occipital lobe), *n* (%)	4 (2.09)	2 (1.32)	2 (5.00)	0.85	0.194
Location (insular lobe), *n* (%)	11 (5.76)	8 (5.30)	3 (7.50)	0.02	0.881
Clinical symptoms, *n* (%)					
Headache	59 (30.89)	39 (25.83)	20 (50.00)	8.66	0.003*
Dizziness	20 (10.47)	14 (9.27)	6 (15.00)	0.58	0.446
Nausea	6 (3.14)	4 (2.65)	2 (5.00)	0.06	0.804
Vomiting	8 (4.19)	5 (3.31)	3 (7.50)	0.54	0.464
Seizure	20 (10.47)	18 (11.92)	2 (5.00)	0.96	0.327
Intracranial space‐occupying lesion or intracranial tumor	6 (3.14)	5 (3.31)	1 (2.50)	0.00	1.000
Impaired consciousness	58 (30.37)	47 (31.13)	11 (27.50)	0.20	0.657
Limb numbness	18 (9.42)	15 (9.93)	3 (7.50)	0.03	0.870
Speech disorder	13 (6.81)	8 (5.30)	5 (12.50)	1.58	0.209
Memory deterioration	3 (1.57)	3 (1.99)	0 (0.00)	0.00	1.000
Visual impairment	5 (2.62)	5 (3.31)	0 (0.00)	0.37	0.542
Behavioral data, mean ± SD					
KPS	95.76 ± 6.10	96.09 ± 6.11	94.50 ± 5.97	1.47	0.142
MMSE	26.29 ± 3.46	26.62 ± 3.19	25.07 ± 4.15	2.54	0.012*
AQ	92.42 ± 7.12	93.35 ± 6.17	88.92 ± 9.19	2.88	0.006*
CQ	92.97 ± 8.63	93.90 ± 7.53	89.45 ± 11.36	2.35	0.023*
Spontaneous speech	90.63 ± 8.64	91.52 ± 8.06	87.25 ± 9.93	2.83	0.005*
Conversational question	94.24 ± 8.42	87.68 ± 11.34	84.50 ± 11.31	1.58	0.116
Personal description	87.02 ± 11.38	95.36 ± 7.28	90.00 ± 10.86	2.95	0.005*
Repetition	96.35 ± 7.22	97.37 ± 5.53	92.47 ± 10.82	2.77	0.008*
Sentences repetition	95.17 ± 9.48	96.52 ± 7.24	90.08 ± 14.24	2.77	0.008*
Naming	90.81 ± 9.08	91.73 ± 8.13	87.33 ± 11.48	2.77	0.006*
Object naming	95.68 ± 6.78	96.38 ± 5.52	93.03 ± 9.86	2.07	0.044*
Responsive speech	94.19 ± 10.12	94.44 ± 9.21	93.25 ± 13.09	0.66	0.511
Word fluency	73.04 ± 23.05	75.30 ± 22.00	64.50 ± 25.14	2.68	0.008*
Auditory verbal comprehension	93.78 ± 8.49	94.74 ± 7.08	90.15 ± 11.88	2.34	0.024*
Yes/No questions	93.86 ± 7.30	94.31 ± 6.96	92.17 ± 8.32	1.65	0.100
Auditory recognition	96.82 ± 5.02	97.19 ± 4.53	95.42 ± 6.44	1.99	0.048*
Sequential commands	90.28 ± 17.17	92.25 ± 14.29	82.85 ± 24.09	2.36	0.023*
Reading	94.57±10.41	95.36 ± 9.87	91.60 ± 11.89	1.84	0.071
Reading words	94.50±7.72	97.62 ± 10.56	94.75 ± 14.32	1.41	0.161
Spoken word‐written word choice matching	97.02±11.47	97.45 ± 5.65	97.00 ± 5.64	0.45	0.655
Written word stimulus‐picture choice matching	97.95 ± 5.27	98.58±6.62	97.88±5.76	0.61	0.542
Read out	97.36 ± 5.64	96.19 ± 14.14	97.83 ± 6.32	−0.71	0.476
Comprehension	98.43 ± 6.44	93.81 ± 17.95	88.03 ± 23.34	1.70	0.091
Reading commands	94.60 ± 15.20	91.93 ± 17.89	82.53 ± 26.84	2.10	0.041*
Read out	96.53 ± 12.90	97.62 ± 10.56	94.75 ± 14.32	1.41	0.161
Comprehension	92.60 ± 19.28	97.45 ± 5.65	97.00 ± 5.64	0.45	0.655
Reading comprehension of sentences	89.96 ± 20.38	98.58 ± 6.62	97.88 ± 5.76	0.61	0.542
Writing	89.08 ± 13.82	90.11 ± 12.05	85.17 ± 18.78	1.58	0.121
Writing on request	96.28 ± 10.48	96.16 ± 10.82	96.75 ± 9.17	−0.32	0.752
Copying a sentence	96.81 ± 10.89	96.89 ± 10.53	96.50 ± 12.31	0.20	0.842
Series writing	99.42 ± 7.53	100.00^a^	96.75 ± 16.03	1.33	0.190
Writing to dictation	88.27 ± 17.64	89.64 ± 14.78	83.08 ± 25.30	1.57	0.123
Written output	77.51 ± 28.06	79.04 ± 26.48	71.75 ± 33.12	1.47	0.145
Spontaneous writing	72.88 ± 35.06	74.97 ± 34.04	65.00 ± 38.10	1.61	0.110

*Note*: *p <* 0.05 was considered statistically significant and marked with an asterisk (*), *p < 0.05, **p < 0.001. Continuous data are shown as mean ± SD, with statistical significance based on the two‐sample *t*‐test. Categorical data differences (No. and percentages) are represented with statistical significance based on the chi‐squared test (χ ^2^ & *p*) and Fisher's exact test (z & *p*). Language scores have been normalized into percentages based on their full marks, which refer to mini‐mental state examination and the Aphasia Battery of Chinese (ABC), which was adapted from the Western Aphasia Battery (WAB). The scores have been normalized to percentages relative to the maximum attainable score for each test. Spontaneous speech (full marks: 200), repetition (full marks: 200), naming (full marks: 300), auditory verbal comprehension (full marks: 300), reading (full marks: 900), writing (full marks: 600). Behavioral data were obtained for the day before the surgery, and all patients underwent an assessment which performed by an experienced neuropsychologist.

Abbreviations: AQ = Aphasia Quotient; CQ = Cortical Quotient; KPS = Karnofsky performance status; MMSE = mini‐mental state examination; PFS = progression‐free survival.

^a^ All patients without recurrence have achieved perfect scores in series writing. All patients have achieved perfect scores in word repetition.

To assess the characteristics in patients with different molecular parameters, demographic, clinical, and behavioral data of patients for different statuses of IDH1/2 mutation (*n*  =  188), MGMT promoter methylation (*n*  =  60), and 1p/19q codeletion (*n*  =  70) are presented in Tables S2–S4. For IDH1/2 mutation, the IDH‐mutant group were younger than IDH‐wildtype group (41.71 [9.95] vs. 45.13 [11.08] years, *t* = −2.17, *p* = 0.031), Gliomas located in the temporal and parietal lobe were predominantly observed in the IDH‐wildtype group (9.09% vs. 23.88%, χ ^2^ = 7.67, *p* = 0.006; 3.31% vs. 16.42%, χ ^2^ = 10.10, *p* = 0.001), while the proportion of glioma located in the frontal lobe was higher in the IDH‐mutant group (53.72% vs. 31.34%, χ ^2^ = 8.70, *p* = 0.006). The speech disorder was more frequently observed in the IDH‐wildtype group (1.65% vs. 16.42%, χ ^2^ = 12.40, *p* < 0.001). For MGMT promoter methylation, the proportion of glioma located in the insular lobe was higher in patients with MGMT promoter methylation (22.22% vs. 0.00%, *z* = 7.82, *p* = 0.020). As for 1p/19q codeletion, the proportion of WHO 2 was lower in patients with 1p/19q‐noncodeletion (52.08% vs. 86.36%, *z* = 7.59, *p* = 0.006).

In the aspect of behavioral scores, the group of patients with IDH1/2 mutation achieved higher scores in KPS, MMSE, AQ, CQ, and language tests of spontaneous speech, repetition, naming, auditory verbal comprehension, reading, and writing. There were no significant differences between MGMT promoter methylation and 1p/19q codeletion status. Detailed characteristics of scores and corresponding statistical results were summarized in Tables S2–S4.

### 3.2 Survival Analyses

Univariable Cox proportional‐hazards (CPH) model analyses revealed a total of 17 significant predictors related to 12‐month recurrence (Table 2). Principal component analysis (PCA) was performed on the significant language score items, considering the correlation between language scores. The cumulative contribution percentage of the first five components reached 88.3%, and the components were named according to the absolute value of the coefficient of the index in each component (Table S5). These components were then incorporated into the multivariate Cox model; the factors of tumor location were treated as dummy variates, and the occipital lobe was set as the reference (Table 2). The log‐rank test indicated no statistically significant differences in survival curves between the recurrence risk groups (χ ^2^ = 14.46, *p* = 0.273).

**TABLE 2:** Univariate and multivariate analyses of factors associated with recurrence.

Variables	Univariable Cox regression		Multivariable Cox regression	
	HR (95%CI)	*p*‐value	HR (95%CI)	*p*‐value
Age at diagnosis (years)	1.02 (0.98–1.07)	0.234	0.97 (0.92–1.03)	0.306
Sex (male)	1.56 (0.64–3.84)	0.329	1.25 (0.44–3.5)	0.677
Education (years)	1.09 (0.96–1.23)	0.188	1.12 (0.97–1.3)	0.118
Location (frontal lobe)	0.25 (0.08–0.73)	0.012*	0.19 (0.05–0.7)	0.012*
Location (temporal lobe)	1.78 (0.65–4.82)	0.259	0.51 (0.11–2.42)	0.397
Location (parietal lobe)	1.76 (0.60–5.22)	0.306	0.54 (0.08–3.51)	0.516
Location (occipital lobe)^a^	1.68 (0.22–12.48)	0.614		
Location (insular lobe)	1.58 (0.37–6.79)	0.535	0.60 (0.06–5.78)	0.658
Headache	1.65 (0.71–3.83)	0.245		
Nausea	2.48 (0.33–18.56)	0.376		
Vomiting	3.88 (0.90–16.73)	0.069		
Epilepsy	1.28 (0.30–5.50)	0.743		
Intracranial space‐occupying lesion or intracranial tumor	1.63 (0.22–12.13)	0.634		
Impaired consciousness	0.91 (0.36–2.34)	0.850		
Speech disorder	1.94 (0.45–8.35)	0.372		
KPS	0.94 (0.88–1.01)	0.108	0.92 (0.84–1.01)	0.065
MMSE	0.89 (0.81–0.99)	0.027*		
AQ	0.93 (0.89–0.98)	0.006*		
CQ	0.95 (0.91–0.98)	0.005*		
Spontaneous speech	0.96 (0.92–1.01)	0.111		
Conversational question	0.99 (0.95–1.02)	0.484		
Personal description	0.95 (0.91–0.99)	0.022*		
Repetition	0.95 (0.92–0.98)	0.002*		
Sentences repetition	0.96 (0.94–0.99)	0.002*		
Naming	0.95 (0.91–0.99)	0.015*		
Object naming	0.94 (0.90–0.99)	0.018*		
Responsive speech	0.98 (0.94–1.01)	0.210		
Word fluency	0.98 (0.97–1.00)	0.070		
Auditory verbal comprehension	0.95 (0.92–0.98)	0.003*		
Yes/No questions	0.96 (0.91–1.00)	0.060		
Auditory recognition	0.92 (0.86–0.98)	0.012*		
Sequential commands	0.98 (0.96–0.99)	0.006*		
Reading	0.98 (0.95–1.01)	0.118		
Reading words	1.01 (0.95–1.07)	0.735		
Spoken word‐written word choice matching	0.98 (0.95–1.00)	0.060		
Written word stimulus‐picture choice matching	0.96 (0.91–1.01)	0.130		
Read out	0.97 (0.92–1.03)	0.338		
Comprehension	0.96 (0.92–1.01)	0.113		
Reading commands	0.99 (0.97–1.01)	0.310		
Read out	1.01 (0.97–1.06)	0.618		
Comprehension	0.98 (0.97–1.00)	0.049*		
Reading comprehension of sentences	0.99 (0.97–1.00)	0.048*		
Writing	0.98 (0.95–1.00)	0.035*		
Writing on request	1.01 (0.96–1.05)	0.764		
Copying a sentence	1.00 (0.96–1.06)	0.861		
Series writing	0.97 (0.95–1.00)	0.019*		
Writing to dictation	0.98 (0.96–1.00)	0.034*		
Written output	0.99 (0.98–1.00)	0.075		
Spontaneous writing	1.00 (0.98–1.01)	0.436		
Principal components				
Auditory verbal comprehension & writing^b^			0.8 (0.68–0.94)	0.007*
Repetition^b^			1.31 (1.01–1.71)	0.040*
Series writing & sequential commands^b^			0.98 (0.74–1.29)	0.873
Naming^b^			0.91 (0.6–1.39)	0.670
Personal description & series writing^b^			0.68 (0.37–1.24)	0.205

*Note*: *p <*0.05 was considered statistically significant and marked with an asterisk (*), *p < 0.05, **p < 0.001.

Abbreviations: AQ = Aphasia Quotient; CI = confidence interval; CQ = Cortical Quotient; HR = Hazard ratio; KPS = Karnofsky performance status; MMSE = mini‐mental state examination.

^a^ The factors of tumor location were treated as dummy variates, and the occipital lobe was set as the reference.

^b^ Considering the correlation among language scores, principal component analysis was conducted on all significant language scores in univariate Cox analysis. The top five components, which collectively contributed to 88.3% of the variance, were selected to represent the language scores in the multivariable Cox model. The principal components were named according to the two indices with the largest coefficients.

**FIGURE 2:**
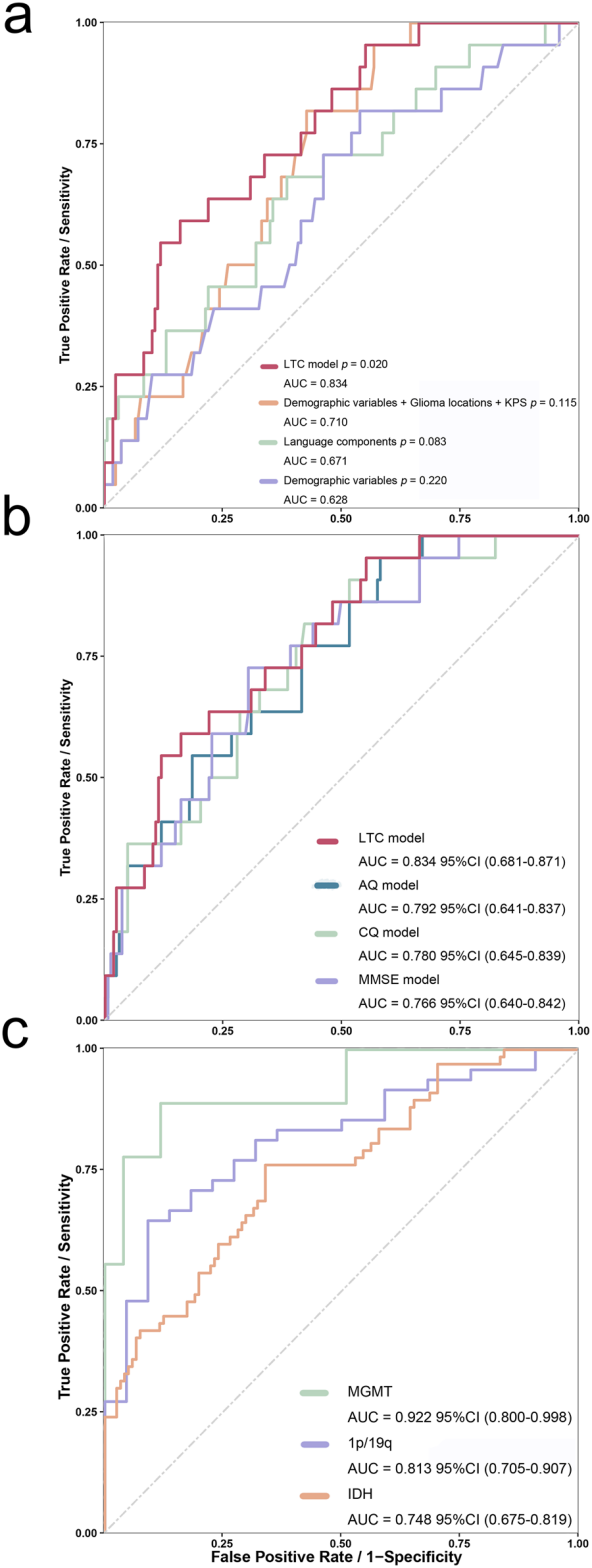
**ROC curves generated for the preoperative predictors**. (A) ROC curves comparing the predictive performance of four preoperative models constructed by incremental inclusion of predictors. The models include a demographics‐only model (age at diagnosis, sex, and education), a language‐components model (five language variables), a demographics + glioma location + KPS model, and the LTC model incorporating demographics, KPS, glioma locations, and language components. The language components include auditory verbal comprehension, repetition, writing, naming, and spontaneous speech. The glioma location variables include the frontal lobe, temporal lobe, parietal lobe, and insular lobe. The progressive improvement in AUC illustrates the added predictive value of language assessments. (B) ROC curves are utilized to depict the predictive performance of different models. The LTC model (AUC, 0.834; 95% Cl, 0.681–0.871) compared with the AQ model (AUC,0.792; 95% Cl, 0.641–0.837), CQ model (AUC,0.780; 95% Cl, 0.645–0.839), and MMSE model (AUC, 0.766; 95% Cl, 0.640–0.842). (C) ROC curves are utilized to illustrate the predictive capability of the LTC model for different pathological molecular parameters. The MGMT (AUC, 0.922; 95% Cl, 0.800–0.998), the 1p/19q (AUC, 0.813; 95% Cl, 0.705–0.907), and the IDH1 (AUC, 0.748; 95% Cl, 0.675–0.819), respectively. Demographic variables included age at diagnosis, sex, and education level. Glioma locations included location: frontal lobe, location: temporal lobe, and location: parietal lobe. Language components included auditory verbal comprehension & writing, repetition, series writing & sequential commands, naming, and personal description & series writing. LTC model = language tests combinations model; KPS = Karnofsky performance status; MMSE = mini‐mental state examination; AQ = Aphasia Quotient; CQ = Cortical Quotient.

ROC curves were employed to evaluate the sensitivity and specificity of the model. To illustrate the contribution of language scores to the improvement of predictive performance and to compare the language‐based model with other commonly used prediction models and to benchmark its performance against a clinically relevant baseline, we incrementally incorporated predictors and plotted ROC curves for four models: the demographics‐only model (AUC = 0.628, *p* = 0.220), the language‐components model (AUC = 0.671, *p* = 0.083), the demographics + glioma location + KPS model (AUC = 0.710, *p* = 0.115), and the language tests combinations (LTC) model (AUC = 0.834, *p* = 0.020) (Figure 2A). Given the limited sample size, these AUCs should be interpreted as exploratory upper‐bound estimates rather than indicators of clinical‐grade predictive performance. The data of the LTC model were resampled 3000 times using the Bootstrap method, and the mean AUC for resampling remained 0.834, with a 95% confidence interval of (0.805, 0.863) (Figure S1). To further evaluate the temporal stability and longer‐term clinical relevance of the LTC model, time‐dependent ROC analyses were additionally performed at 24 months and over the entire follow‐up period (8 years). The model achieved an AUC of 0.813 (*p* = 0.007; 95% CI, 0.648–0.831) at 24 months and 0.748 (*p* < 0.001; 95% CI, 0.648–0.821) over the full follow‐up (Figure S2). To assess the robustness of model performance with respect to the number of retained principal components, principal components were sequentially incorporated into the model according to the order of cumulative contribution percentage derived from the PCA. Models including one to five principal components were constructed, with the corresponding AUCs and *p*‐values summarized in Table S6. The LTC derived from the multivariable CPH model was replaced with the other behavioral scores of AQ, CQ, and MMSE, respectively (Figure 2B). To appraise the predicted ability of molecular parameters by LTC model, ROC curves showed the stronger predicted ability in MGMT promoter methylation (AUC, 0.922; 95%Cl, 0.800–0.998) and 1p/19q codeletion (AUC, 0.813; 95%Cl, 0.705–0.907), and in IDH1/2 mutation (AUC, 0.748; 95%Cl, 0.675–0.819) (Figure 2C).

### 3.3 Language Tests Combinations Model

**FIGURE 3:**
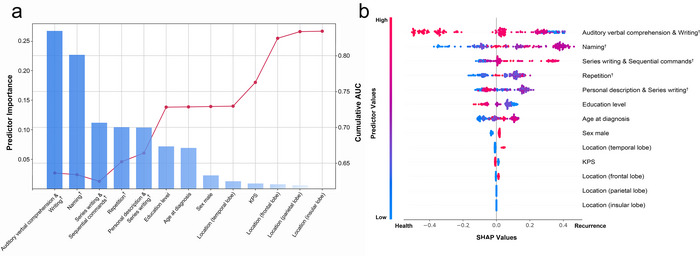
**Predictors selection and SHAP visualization of modelling on glioma patients**. (A) Sequential forward selection from preselected candidate predictors. The bar chart represented the sorted predictor importance based on their contributions to model classifications. The line chart delineated cumulative AUCs (left axis) upon the inclusion of predictors one by each iteration. (B) SHAP visualization plot of selected predictors. The width of the range of horizontal bars can be interpreted as the impact on the model prediction that the wider its range, the larger its impact. The color of the horizontal bars represented the magnitude of predictors, which was coded in a gradient from blue (low) to red (high), shown as the color bar on the right‐hand side. The directions on the x‐axis represented the likelihood of recurrence (right) or health (left). Readers can then infer the possibility of recurrence, given each predictor's specific value (coded in a gradient of colors). LTC model = language tests combinations model; KPS = Karnofsky performance status.

Figure 3 illustrates the contribution of each language component to the recurrence prediction model, as revealed by SHAP analysis. The predictors ultimately included in the LTC model are ranked according to their importance in the prediction task, as depicted in the bar chart (Figure 3A). the auditory verbal comprehension & writing, naming, series writing & sequential commands, repetition and personal description & series writing were considered to be the predictors that contributed the most to the predictive performance of the model. The sequential forward selection scheme is illustrated by a line chart, demonstrating that as the first few predictors are incorporated, the model's performance (AUC on the right axis) gradually increases, reaching its peak as the predictor with the smallest contribution (glioma located at the frontal lobe) is ultimately included.

Additionally, the Beeswarm plot summarized the importance of predictors in the LTC model (Figure 3B). Each patient exhibited as a data point, and the color of the horizontal bars represented the magnitude of predictors, which were coded in a gradient from blue (low) to red (high), shown as the color bar on the left‐hand side. The directions on the *x*‐axis represented the likelihood of recurrence (right) or being healthy (left). The possibility of recurrence given each predictor's specific value (coded in a gradient of colors) could be inferred. The width of the range of horizontal bars can be interpreted as the impact on the model prediction, that the wider its range, the larger its impact. Apparently, auditory verbal comprehension & writing had the widest range, indicating the most considerable prediction power and impacting the model's output.

Nomograms were developed using the LTC model to estimate the probability of 12‐month glioma recurrence (Figure S3). To calculate a patient's 12‐month glioma recurrence probability with a nomogram, one must first locate the patient's position on each variable axis of the nomogram based on their individual characteristics or clinical parameters. Once the patient's position on each axis is determined, a straight line is drawn from each variable axis to the point axis. The points obtained from each variable are then summed to derive a total points value. This total points value is then located on the total points axis, and a straight line is drawn downwards to intersect with the probability axis, thus providing the estimated 12‐month glioma recurrence probability for the patients.

## 4 Discussion

The preoperative prediction of glioma prognosis holds significant importance in guiding treatment decisions and postoperative rehabilitation. This study analyzed a cohort of 191 glioma patients to develop a model for predicting 12‐month glioma recurrence based on preoperative language functions. Significant predictors identified through univariable and multivariable Cox proportional‐hazards analyses included scores of auditory verbal comprehension & writing, naming, series writing & sequential commands, repetition, and personal description & series writing. The LTC model demonstrated robust performance in predicting 12‐month glioma recurrence, with an AUC of 0.834. Furthermore, this model also showed potential in preoperative detection of key molecular parameters, including IDH1/2 mutation, MGMT promoter methylation, and 1p/19q codeletion. The nomogram based on the predictive model provided clinicians with a convenient prognostic assessment tool, aiding in optimizing clinical risk management. These findings underscore the importance of integrating language function assessments into glioma prognosis to evaluate risk factors and adjust treatment and rehabilitation strategies accordingly.

Compared with models developed using sophisticated multimodal neuroimaging or invasive histopathological examinations, our model primarily relies on easily obtainable predictors derived from standardized language assessments. Although task‐based fMRI and MEG can provide valuable information on the functional organization of language networks, their clinical applicability remains limited. fMRI activation patterns, while generally consistent with results from direct cortical stimulation, cannot independently determine aphasia type, as patients with similar imaging features may present with different linguistic deficits (Brannen et al. 2001). MEG, despite its excellent temporal resolution, requires prolonged head fixation and specialized magnetic‐shielding environments, which significantly restrict its use in routine clinical practice. Consequently, comprehensive linguistic evaluation remains indispensable and cannot be replaced by neuroimaging alone. Given these considerations, the LTC model, which is built upon brief, accessible, and validated language assessments, has the potential to be implemented across medical institutions with varying levels of resources and therefore supports broader clinical utility. Previous studies have established age, sex, and KPS as clinically relevant predictors of glioma recurrence (Lacroix et al. 2001; Nahm 2022; Thakkar et al. 2014; van Kessel et al. 2021). This study provides novel insights by exploring language function as a predictor of glioma prognosis. Preoperative language impairments are common in glioma patients and are often attributed to tumor invasion of widely distributed language areas (Brown et al. 2013; De Witt Hamer et al. 2012). Moreover, the repeated tumor progression in recurrent glioma could cause severe preoperative language deficits, especially in naming and fluency (Hardy et al. 2016; Wefel et al. 2011), which is consistent with our study. Besides, auditory verbal comprehension was considered to be an important score for responding to early postoperative language function (Wakamatsu et al. 2023), while the writing impairment often implied a poor prognosis (Guarracino et al. 2021). In our previous studies, the language function, including auditory verbal comprehension, naming, and fluency, demonstrated a complex brain functional network involving numerous brain regions (Sun et al. 2024; J. Zhang et al. 2023). Therefore, as key predictors in the prediction model of this study, language predictors could generally reflect the extent of damage caused by tumor invasion. Our results align with existing literature on the impact of glioma location on language function but extend these findings by quantifying the predictive value of specific language impairments (De Witt Hamer et al. 2012). These findings suggest that language dysfunction may serve as a potent predictor of recurrence in glioma patients.

In this study, the LTC model incorporated language scores using PCA to address collinearity among correlated language measures. The number of retained components was determined by balancing dimensionality reduction with the preservation of clinically relevant information, following commonly adopted practices in PCA applications that aim to capture the majority of variance while maintaining model interpretability and stability. Overall, model discrimination improved as additional principal components were included, indicating that progressively capturing more informative variance in language function enhanced predictive performance. Notably, the similarity in AUC values between the models incorporating four and five principal components suggests a performance plateau, supporting the stability of the LTC model and mitigating concerns regarding overfitting (Table S6). These results indicate that the observed predictive performance is not driven by an arbitrary dimensionality reduction threshold, but reflects a consistent and robust contribution of language‐related variance to outcome prediction while maintaining an appropriate balance between model complexity and sample size. Discrimination metrics such as AUC should be interpreted cautiously in the context of sparse events and are more informative for relative model comparison than absolute predictive accuracy. The model's sensitivity and specificity were evaluated using ROC curves and validated internally using the Bootstrap method, which was one of the TRIPOD statement reporting recommendations of internal validation (Collins et al. 2024). The sample size of this study was constrained by the availability of matched behavioral data, resulting in a relatively limited dataset. The Bootstrap method addressed this by generating multiple samples through repeated random sampling with replacement. This approach improved the model's stability and reliability by mitigating sampling bias and reducing the risk of overfitting. By providing robust performance estimates, it supported a more accurate assessment of the model's generalizability. Compared with models based on demographic data, glioma locations, and the other behavioral scores, including AQ, CQ, and MMSE, the LTC model demonstrated superior predictive performance with an AUC of 0.834. To further evaluate the temporal stability and broader applicability of the LTC model, time‐dependent ROC analyses were additionally performed at 24 months and over the entire follow‐up period (8 years). The findings demonstrated good short‐term predictive ability and sustained, stable long‐term predictive performance. This result illustrated a strong correlation between selected predictors and recurrence of glioma. The study employed SHAP to interpret the importance of predictors in the LTC model. Auditory verbal comprehension & writing, naming, series writing & sequential commands, repetition and personal description & series writing emerged as key language predictors, as shown in bar and line charts. These findings reaffirmed that glioma recurrence is associated with functional status (Kelly et al. 2017). The nomograms derived from the LTC model offer a practical tool for estimating the probability of 12‐month glioma recurrence, thereby assisting clinicians in prognosis assessment.

As a novel attempt, our study aimed to explore the relationship between pathological molecular subtypes of glioma and preoperative language function scores. In the fifth edition of the World Health Organization 2021 CNS classification (WHO CNS5), IDH1/2 is crucial in distinguishing between low‐grade gliomas (LGG) and high‐grade gliomas (HGG), which is essential for determining the appropriate therapeutic approach (Qian et al. 2018; Suh et al. 2019). IDH‐mutant gliomas include astrocytomas and oligodendrogliomas; when accompanied by 1p/19q codeletion, they are classified as oligodendrogliomas with a relatively favorable prognosis, whereas IDH‐wildtype gliomas are generally more aggressive and often correspond to glioblastoma (Boots‐Sprenger et al. 2013; Li et al. 2016; Louis et al. 2021; McNamara et al. 2022). In addition, MGMT promoter methylation remains an important predictive biomarker for responsiveness to alkylating chemotherapy, particularly temozolomide, especially in IDH‐wildtype gliomas (Kitange et al. 2009). The molecular profiles could largely influence the surgical decision‐making (Xiong et al. 2022). For IDH‐mutant gliomas, maximal safe resection should be performed to improve PFS and overall survival (OS) (Hou et al. 2021). In contrast, for those without IDH mutations, a more aggressive supratotal resection could be adopted (De Leeuw and Vogelbaum 2019). However, for patients with IDH mutation and 1p/19q codeletion, a subtotal resection could also be acceptable since the higher sensitivity to radiotherapy and chemotherapy (Wijnenga et al. 2018). Therefore, the protection of brain functions ranks higher than the total resection of the tumor.

As the molecular biomarkers can only be definitively determined postoperatively, and the turnaround time varies across institutions, we explored preoperative predictors of molecular pathology. In this study, we used the LTC model to perform logistic regression analyses for IDH1/2 mutation, MGMT promoter methylation, and 1p/19q codeletion. Logistic regression estimates the probability of a tumor carrying a specific molecular feature based on input variables, and ROC curves were used to evaluate the predictive performance of the derived models. The results demonstrated good predictive ability across all three molecular markers, suggesting that language‐based measures may carry useful information for molecular subtype inference.

However, the observed associations may reflect indirect effects mediated by tumor location, grade, and neuroanatomical involvement, rather than a direct biological linkage between language function and molecular alterations. Several mechanisms may explain the observed associations between preoperative language function and molecular status. Different molecular subtypes exhibit distinct biological behaviors, growth kinetics, and anatomical predilections, which may lead to varying impacts on language‐related cortical and subcortical networks. For example, IDH‐mutant and 1p/19q‐codeleted oligodendrogliomas tend to grow slowly, allowing greater opportunity for functional compensation within the language network, potentially resulting in milder impairment. In contrast, the more infiltrative and rapidly progressive IDH‐wildtype tumors or MGMT‐unmethylated gliomas may produce more pronounced and acute language deficits. Moreover, molecular alterations may influence tumor metabolism, white‐matter tract involvement, and local microenvironmental changes, all of which are relevant to language processing networks. Recent evidence also indicates that molecular markers such as IDH mutations are associated with preoperative cognitive performance in glioma patients (K. Zhang et al. 2024), supporting the plausibility of molecular–cognitive interactions.

### 4.1 Limitations

This study has some limitations that should be considered when interpreting the findings. First, as a single‐center retrospective analysis, the study is inherently subject to selection bias and potential residual recall bias, although all behavioral assessments were obtained one day before surgery to minimize this risk. The exclusion of patients with incomplete records may also have introduced systematic differences between included and excluded individuals, thereby limiting the generalizability of the results. In addition, the absence of a multicenter cohort and external validation restricts the model's transferability to broader clinical settings; internal validation alone cannot fully assess its generalization performance, although bootstrap validation is a reliable approach recognized by TRIPOD for validating model stability on existing data. In addition, calibration analysis and decision curve analysis, which are particularly informative for individualized risk communication and threshold‐based clinical decision‐making, were not performed in the current study due to the retrospective design and the absence of clearly defined intervention thresholds, and will be prioritized in future prospective and multicenter validations. We are currently integrating institutional resources and actively establishing collaborations with additional medical centers to enable multicenter data collection and external validation in future research, as well as considering prospective study designs to strengthen causal inference. Furthermore, the availability of molecular marker data was limited: MGMT promoter methylation and 1p/19q codeletion were available only for a subset of patients, and key markers such as TERT, EGFR, and BRAF were inconsistently included during the 2010–2018 study period due to the evolving World Health Organization classification criteria for glioma, preventing comprehensive retrospective extraction. This constraint reduced the sample size available for molecular prediction analyses and may have affected the stability of the model, raising the possibility of overfitting. Future studies with larger cohorts and more complete molecular profiles will be essential for building more robust and comprehensive preoperative prediction tools. Another limitation concerns imaging data: tumor volume and extent of resection are well‐established predictors of progression‐free survival and are known to influence language function, but high‐quality MRI scans were not consistently available for the entire cohort, precluding their inclusion in the present analyses. We are currently consolidating imaging resources to incorporate tumor volumetrics in subsequent research, which we believe will further improve model performance. Despite these limitations, the present study provides preliminary evidence supporting the prognostic relevance of language function and lays important groundwork for future multicenter, prospective, and data‐rich investigations.

## 5 Conclusions

In this retrospective cohort study, we developed an exploratory language‐based prognostic model to investigate the association between preoperative language dysfunction and glioma recurrence after surgical resection. Auditory verbal comprehension & writing, naming, series writing & sequential commands, repetition, and personal description & series writing emerged as key language‐related contributors and showed notable associations with molecular parameters. These findings support the hypothesis that language function captures clinically relevant information related to tumor behavior and recurrence risk. Rather than serving as a standalone clinical decision tool, the LTC model provides a hypothesis‐generating and interpretable framework that may complement established prognostic factors and guide future multicenter, prospective studies aimed at refining individualized risk assessment in glioma patients.

## Author Contributions

Acquisition of data, concept and design, statistical analysis, interpretation of results, writing of the manuscript draft: H.S. Concept and design, writing of the manuscript draft, critical revision of the article: L.B. Acquisition of data and interpretation of data: C.L., L.Y., and S.W. Critical revision of the article, approval of the submitted article: J.Z. Concept and design, critical revision of the article, approval of the submitted article: Y.Y.

## Funding

This work is supported by the STI 2030—Major Projects No. 2022ZD0209900, the Innovation Program of Shanghai Municipal Education Commission (2023ZKZD13), the National Natural Science Foundation of China (no. 82327807), and the Zhejiang Provincial Natural Science Foundation of China (no. LQ23H090008).

## Disclosure

An unauthorized version of the Chinese MMSE was used by the study team without permission; however, this has now been rectified with PAR. The MMSE is a copyrighted instrument and may not be used or reproduced in whole or in part, in any form or language, or by any means without the written permission of PAR (www.parinc.com).

## Ethics Statement

The study protocol was approved by the ethics review committee of Huashan Hospital Affiliated to Fudan University (number KY2015‐256) and was registered in the project Molecular Pathology Research Project of Glioma (NCT04924127, https://clinicaltrials.gov/show/NCT04924127). Written informed consents were obtained from all patients.

## Conflicts of Interest

The authors declare no conflicts of interest.

## Consent to Publish

Not applicable.

## Supporting information



Table S1 Items and scoring of the Aphasia Battery of Chinese (ABC).
Table S2 Baseline characteristics of glioma patients in idh1/2 status.
Table S3 Baseline characteristics of glioma patients in MGMT status.
Table S4 Baseline characteristics of glioma patients in 1p/19q status.
Table S5 Principal component analysis (PCA) on language test scores.
Table S6 Predictive performance of models incorporating different numbers of principal components.
Figure S1 AUC distribution and quantile plots for the LTC model based on bootstrap.
Figure S2 Time‐dependent ROC analyses of the LTC model at different prediction horizons.
Figure S3 Nomogram for predicted 12‐month recurrence probability in glioma patients.

## Data Availability

The datasets used and analyzed during the current study are available from the corresponding author on reasonable request.

## References

[brb371243-bib-0001] Abbafati, C. , K. M. Abbas , M. Abbasi , et al. 2020. “Global Burden of 369 Diseases and Injuries in 204 Countries and territories, 1990–2019: A Systematic Analysis for the Global Burden of Disease Study 2019.” Lancet 396, no. 10258: 1204–1222. 33069326 10.1016/S0140-6736(20)30925-9 PMC7567026

[brb371243-bib-0002] Abdi, H. , and L. J. Williams . 2010. “Principal Component Analysis.” WIREs Computational Statistics 2, no. 4: 433–459. 10.1002/wics.101.

[brb371243-bib-0003] Alhasan, A. S. n.d. “Clinical Applications of Artificial Intelligence, Machine Learning, and Deep Learning in the Imaging of Gliomas: A Systematic Review.” Cureus 13, no. 11: e19580. 10.7759/cureus.19580. PMC8671075 34926051

[brb371243-bib-0004] Boots‐Sprenger, S. H. E. , A. Sijben , J. Rijntjes , et al. 2013. “Significance of Complete 1p/19q Co‐Deletion, IDH1 Mutation and MGMT Promoter Methylation in Gliomas: Use With Caution.” Modern Pathology 26, no. 7: 922–929. 10.1038/modpathol.2012.166. 23429602

[brb371243-bib-0005] Brannen, J. H. , B. Badie , C. H. Moritz , M. Quigley , M. E. Meyerand , and V. M. Haughton . 2001. “Reliability of Functional MR Imaging With Word‐Generation Tasks for Mapping Broca's Area.” AJNR American Journal of Neuroradiology 22, no. 9: 1711–1718. 11673166 PMC7974431

[brb371243-bib-0006] Brown, T. , A. H. Shah , A. Bregy , et al. 2013. “Awake Craniotomy for Brain Tumor Resection: The Rule Rather Than the Exception?” Journal of Neurosurgical Anesthesiology 25, no. 3: 240–247. 10.1097/ANA.0b013e318290c230. 23603885

[brb371243-bib-0007] Collins, G. S. , P. Dhiman , J. Ma , et al. 2024. “Evaluation of Clinical Prediction Models (Part 1): From Development to External Validation.” Bmj 384: e074819. 10.1136/bmj-2023-074819. 38191193 PMC10772854

[brb371243-bib-0008] De Leeuw, C. N. , and M. A. Vogelbaum . 2019. “Supratotal Resection in Glioma: A Systematic Review.” Neuro‐Oncology 21, no. 2: 179–188. 10.1093/neuonc/noy166. 30321384 PMC6374756

[brb371243-bib-0009] De Witt Hamer, P. C. , S. G. Robles , A. H. Zwinderman , H. Duffau , and M. S. Berger . 2012. “Impact of Intraoperative Stimulation Brain Mapping on Glioma Surgery Outcome: A Meta‐Analysis.” Journal of Clinical Oncology 30, no. 20: 2559–2565. 10.1200/jco.2011.38.4818. 22529254

[brb371243-bib-0010] Duffau, H. , and L. Capelle . 2004. “Preferential Brain Locations of Low‐Grade Gliomas.” Cancer 100, no. 12: 2622–2626. 10.1002/cncr.20297. 15197805

[brb371243-bib-0011] Evangelou, K. , I. Kotsantis , A. Kalyvas , et al. 2025. “Artificial Intelligence in the Diagnosis and Treatment of Brain Gliomas.” Biomedicines 13, no. 9: 2285. 10.3390/biomedicines13092285. 41007844 PMC12467936

[brb371243-bib-0012] Gao, S. 1992. “A Study on Standardization of Aphasia Battery of Chinese.” China Mental Health Miscellaneous Records 6, no. 3: 125.

[brb371243-bib-0013] Gao, S. , Y. Zhu , S. Shi , Y. Peng , et al. 1992. “Standard Aphasia Battery of Chinese.” Chinese Mental Health Journal 6: 125–128.

[brb371243-bib-0014] Gómez Vecchio, T. , A. Neimantaite , A. Corell , et al. 2021. “Lower‐Grade Gliomas: An Epidemiological Voxel‐Based Analysis of Location and Proximity to Eloquent Regions.” Frontiers in Oncology 11: 748229. 10.3389/fonc.2021.748229. 34621684 PMC8490663

[brb371243-bib-0015] Guarracino, I. , T. Ius , C. Baiano , S. D'Agostini , M. Skrap , and B. Tomasino . 2021. “Pre‐Surgery Cognitive Performance and Voxel‐Based Lesion‐Symptom Mapping in Patients with Left High‐Grade Glioma.” Cancers 13, no. 6: 1467. 10.3390/cancers13061467. 33806837 PMC8004913

[brb371243-bib-0016] Hardy, C. J. D. , A. H. Buckley , L. E. Downey , et al. 2016. “The Language Profile of Behavioral Variant Frontotemporal Dementia.” Journal of Alzheimer's Disease: JAD 50, no. 2: 359–371. 10.3233/JAD-150806. 26682693 PMC4740928

[brb371243-bib-0017] Hou, Z. , K. Zhang , X. Liu , et al. 2021. “Molecular Subtype Impacts Surgical Resection in Low‐Grade Gliomas: A Chinese Glioma Genome Atlas Database Analysis.” Cancer Letters 522: 14–21. 10.1016/j.canlet.2021.09.008. 34517083

[brb371243-bib-0018] Huang, L. , S.‐H. K. Chen , S. Xu , et al. 2021. “Augmentative and Alternative Communication Intervention for in‐patient Individuals with Post‐Stroke Aphasia: Study Protocol of a Parallel‐Group, Pragmatic Randomized Controlled Trial.” TRIALS 22, no. 1: 837. 10.1186/s13063-021-05799-0. 34819130 PMC8611624

[brb371243-bib-0019] Jolliffe, I. T. 2002. Principal Component Analysis, (2nd ed). New York: Springer.

[brb371243-bib-0020] Karnofsky, D. A. , W. H. Abelmann , L. F. Craver , and J. H. Burchenal . 1948. “The Use of the Nitrogen Mustards in the Palliative Treatment of Carcinoma with Particular Reference to Bronchogenic Carcinoma.” Cancer 1, no. 4: 634–656. 10.1002/1097-0142(194811)1:4<634::AID-CNCR2820010410>3.0.CO;2-L.

[brb371243-bib-0021] Kelly, C. , P. Majewska , S. Ioannidis , M. H. Raza , and M. Williams . 2017. “Estimating Progression‐free Survival in Patients With Glioblastoma Using Routinely Collected Data.” Journal of Neuro‐Oncology 135, no. 3: 621–627. 10.1007/s11060-017-2619-1. 28956223 PMC5700233

[brb371243-bib-0022] Kha, Q.‐H. , V.‐H. Le , T. N. K. Hung , and N. Q. K. Le . 2021. “Development and Validation of an Efficient MRI Radiomics Signature for Improving the Predictive Performance of 1p/19q Co‐Deletion in Lower‐Grade Gliomas.” Cancers 13, no. 21: 5398. 10.3390/cancers13215398. 34771562 PMC8582370

[brb371243-bib-0023] Kitange, G. J. , B. L. Carlson , M. A. Schroeder , et al. 2009. “1p/19q.” Neuro‐Oncology 11, no. 3: 281–291. 10.1215/15228517-2008-090. 18952979 PMC2718972

[brb371243-bib-0024] Kong, L. , J. Wu , J. Gao , et al. 2020. “Particle Radiation Therapy in the Management of Malignant Glioma: Early Experience at the Shanghai Proton and Heavy Ion Center.” Cancer 126, no. 12: 2802–2810. 10.1002/cncr.32828. 32167589 PMC7317504

[brb371243-bib-0025] Kudulaiti, N. , Z. Zhou , C. Luo , J. Zhang , F. Zhu , and J. Wu . 2021. “A Nomogram for Individualized Prediction of Overall Survival in Patients With Newly Diagnosed Glioblastoma: A Real‐World Retrospective Cohort Study.” BMC Surgery 21, no. 1: 238. 10.1186/s12893-021-01233-z. 33957923 PMC8101102

[brb371243-bib-0026] Lacroix, M. , D. Abi‐Said , D. R. Fourney , et al. 2001. “A Multivariate Analysis of 416 Patients With Glioblastoma Multiforme: Prognosis, Extent of Resection, and Survival.” Journal of Neurosurgery 95, no. 2: 190–198. 10.3171/jns.2001.95.2.0190. 11780887

[brb371243-bib-0027] Le, V. H. , T. N. T. Minh , Q. H. Kha , and N. Q. K. Le . 2023. “A Transfer Learning Approach on MRI‐Based Radiomics Signature for Overall Survival Prediction of Low‐Grade and High‐Grade Gliomas.” Medical & Biological Engineering & Computing 61, no. 10: 2699–2712. 10.1007/s11517-023-02875-2. 37432527

[brb371243-bib-0028] Li, Y.‐X. , Z. Shi , A. Aibaidula , et al. 2016. “Not all 1p/19q Non‐Codeleted Oligodendroglial Tumors Are Astrocytic.” Oncotarget 7, no. 40: 64615–64630. 10.18632/oncotarget.11378. 27556304 PMC5323103

[brb371243-bib-0029] Liu, D. , and W. Liu . 2023. “Diagnostic Effect of MRI Diffusion‐Weighted Imaging Apparent Diffusion Coefficient Value on Postoperative Recurrence of Brain Glioma.” Alternative Therapies in Health and Medicine AT9342. 37883754

[brb371243-bib-0030] Louis, D. N. , A. Perry , P. Wesseling , et al. 2021. “The 2021 WHO Classification of Tumors of the Central Nervous System: A Summary.” Neuro‐Oncology 23, no. 8: 1231–1251. 10.1093/neuonc/noab106. 34185076 PMC8328013

[brb371243-bib-0031] McNamara, C. , K. Mankad , S. Thust , et al. 2022. “2021 WHO Classification of Tumours of the central Nervous System: A Review for the Neuroradiologist.” Neuroradiology 64, no. 10: 1919–1950. 10.1007/s00234-022-03008-6. 35869291

[brb371243-bib-0032] Mohammadzadeh, I. , B. Hajikarimloo , B. Niroomand , et al. 2025. “Application of Artificial Intelligence in Forecasting Survival in High‐Grade Glioma: Systematic Review and Meta‐Analysis Involving 79,638 Participants.” Neurosurgical Review 48, no. 1: 240. 10.1007/s10143-025-03419-y. 39954167

[brb371243-bib-0033] Mohammadzadeh, I. , B. Niroomand , B. Hajikarimloo , et al. 2025. “Can We Rely on Machine Learning Algorithms as a Trustworthy Predictor for Recurrence in High‐Grade Glioma? A Systematic Review and Meta‐Analysis.” Clinical Neurology and Neurosurgery 249: 108762. 10.1016/j.clineuro.2025.108762. 39884144

[brb371243-bib-0034] Nahm, F. S. 2022. “Receiver Operating Characteristic Curve: Overview and Practical Use for Clinicians.” Korean Journal of Anesthesiology 75, no. 1: 25–36. 10.4097/kja.21209. 35124947 PMC8831439

[brb371243-bib-0035] Nayak, L. , L. M. DeAngelis , A. A. Brandes , et al. 2017. “The Neurologic Assessment in Neuro‐Oncology (NANO) Scale: A Tool to Assess Neurologic Function for Integration into the Response Assessment in Neuro‐Oncology (RANO) Criteria.” Neuro‐Oncology 19, no. 5: 625–635. 10.1093/neuonc/nox029. 28453751 PMC5464449

[brb371243-bib-0036] Ohgaki, H. , and P. Kleihues n.d. Epidemiology and Etiology of Gliomas. Retrieved November 7, 2024, from https://pubmed.ncbi.nlm.nih.gov/15685439/. 10.1007/s00401-005-0991-y 15685439

[brb371243-bib-0037] Ostrom, Q. T. , L. Bauchet , F. G. Davis , et al. 2014. “The Epidemiology of Glioma in Adults: A “State of the Science” Review.” Neuro‐Oncology 16, no. 7: 896. 10.1093/neuonc/nou087. 24842956 PMC4057143

[brb371243-bib-0038] Patel, A. P. , J. L. Fisher , E. Nichols , et al. 2019. “Global, Regional, and National Burden of Brain and Other CNS Cancer, 1990–2016: A Systematic Analysis for the Global Burden of Disease Study 2016.” The Lancet Neurology 18, no. 4: 376–393. 10.1016/S1474-4422(18)30468-X. 30797715 PMC6416167

[brb371243-bib-0039] Qian, Z. , Y. Li , X. Fan , et al. 2018. “Molecular and Clinical Characterization of IDH Associated Immune Signature in Lower‐Grade Gliomas.” Oncoimmunology 7, no. 6: e1434466. 10.1080/2162402X.2018.1434466. 29872572 PMC5980422

[brb371243-bib-0040] Soldatelli, J. S. , I. M. D. Oliveira , M. C. Kneubil , and J. A. P. Henriques . 2022. “Gliomas Molecular Markers: Importance in Treatment, Prognosis and Applicability in Brazilian Health System.” Anais Da Academia Brasileira de Ciências 94, no. 3: e20211075. 10.1590/0001-3765202220211075. 35766600

[brb371243-bib-0041] Suh, C. H. , H. S. Kim , S. C. Jung , C. G. Choi , and S. J. Kim . 2019. “Imaging Prediction of Isocitrate Dehydrogenase (IDH) Mutation in Patients with Glioma: A Systemic Review and Meta‐Analysis.” European Radiology 29, no. 2: 745–758. 10.1007/s00330-018-5608-7. 30003316

[brb371243-bib-0042] Sun, C. , J. Zhang , L. Bu , J. Lu , Y. Yao , and J. Wu . 2024. “A Speech Fluency Brain Network Derived from Gliomas.” Brain Communications 6, no. 3: fcae153. 10.1093/braincomms/fcae153. 38756538 PMC11098038

[brb371243-bib-0043] Tbahriti, H. F. , A. Boukadoum , M. Benbernou , and M. Belhocine . 2025. “Machine Learning and Deep Learning in Glioblastoma: A Systematic Review and Meta‐Analysis of Diagnosis, Prognosis, and Treatment.” Discover Oncology 16: 1492. 10.1007/s12672-025-03303-7. 40773129 PMC12331544

[brb371243-bib-0044] Thakkar, J. P. , T. A. Dolecek , C. Horbinski , et al. 2014. “Epidemiologic and Molecular Prognostic Review of Glioblastoma.” Cancer Epidemiology, Biomarkers & Prevention 23, no. 10: 1985–1996. 10.1158/1055-9965.EPI-14-0275. PMC4185005 25053711

[brb371243-bib-0045] van Kessel, E. , S. Berendsen , A. E. Baumfalk , et al. 2022. “Tumor‐related Molecular Determinants of Neurocognitive Deficits in Patients With Diffuse Glioma.” Neuro‐Oncology 24, no. 10: 1660–1670. 10.1093/neuonc/noac036. 35148403 PMC9527514

[brb371243-bib-0046] van Kessel, E. , I. M. C. Huenges Wajer , C. Ruis , et al. 2021. “Cognitive Impairments Are Independently Associated with Shorter Survival in Diffuse Glioma Patients.” Journal of Neurology 268, no. 4: 1434–1442. 10.1007/s00415-020-10303-w. 33211158 PMC7990824

[brb371243-bib-0047] Vaz‐Salgado, M. A. , M. Villamayor , V. Albarrán , et al. 2023. “Recurrent Glioblastoma: A Review of the Treatment Options.” Cancers 15, no. 17: 4279. 10.3390/cancers15174279. 37686553 PMC10487236

[brb371243-bib-0048] Vecchio, T. G. , I. Ryden , A. Ozanne , et al. 2024. “Global Health Status and Fatigue Score in Isocitrate Dehydrogenase‐Mutant Diffuse Glioma Grades 2 and 3: A Longitudinal Population‐Based Study From Surgery to 12‐month Follow‐Up.” Neuro‐Oncology Practice 11, no. 3: 347–357. 10.1093/nop/npae017. 38737607 PMC11085849

[brb371243-bib-0049] Wakamatsu, K. , S. Ishiai , N. Aihara , S. Kurokawa , Y. Kimura , and N. Mikuni . 2023. “Prediction of Early Postoperative Language Function by Quantitative Evaluation with Visual and Auditory Naming Tasks During Awake Craniotomy for Brain Tumor Resection: Significance of Auditory Naming Task.” Neurologia Medico‐Chirurgica 63, no. 5: 191–199. 10.2176/jns-nmc.2022-0319. 36858633 PMC10241535

[brb371243-bib-0050] Wefel, J. S. , T. Cloughesy , J. L. Zazzali , et al. 2011. “Neurocognitive Function in Patients with Recurrent Glioblastoma Treated with bevacizumab.” Neuro‐Oncology 13, no. 6: 660–668. 10.1093/neuonc/nor024. 21558074 PMC3107095

[brb371243-bib-0051] Wefel, J. S. , K. R. Noll , G. Rao , and D. P. Cahill . 2016. “Neurocognitive Function Varies by IDH1 Genetic Mutation Status in Patients With Malignant Glioma Prior to Surgical Resection.” Neuro‐Oncology 18, no. 12: 1656–1663. 10.1093/neuonc/now165. 27576872 PMC5744240

[brb371243-bib-0052] Wijnenga, M. M. J. , P. J. French , H. J. Dubbink , et al. 2018. “The Impact of Surgery in Molecularly Defined Low‐grade Glioma: An Integrated Clinical, Radiological, and Molecular Analysis.” Neuro‐Oncology 20, no. 1: 103–112. 10.1093/neuonc/nox176. 29016833 PMC5761503

[brb371243-bib-0053] Xiong, Z. , C. Luo , S. Wu , and J. Wu . 2022. “Research Progress of Surgical Strategies for Glioma Based on Intraoperative Rapid Molecular Pathological Detection.” Journal of Clinical Surgery 30, no. 10: 904–908.

[brb371243-bib-0054] Yuan, B. , H. Xie , F. Gong , et al. 2023. “Dynamic Network Reorganization Underlying Neuroplasticity: The Deficits‐Severity‐Related Language Network Dynamics in Patients with Left Hemispheric Gliomas Involving Language Network.” Cerebral Cortex 33, no. 13: 8273–8285. 10.1093/cercor/bhad113. 37005067

[brb371243-bib-0055] Yuan, B. , N. Zhang , J. Yan , J. Cheng , J. Lu , and J. Wu . 2020. “Tumor Grade‐Related Language and Control Network Reorganization in Patients with Left Cerebral Glioma.” Cortex; A Journal Devoted to the Study of the Nervous System and Behavior 129: 141–157. 10.1016/j.cortex.2020.04.015. 32473401

[brb371243-bib-0056] Zhang, J. , Y. Yao , J.‐S. Wu , et al. 2023. “The Cortical Regions and White Matter Tracts Underlying Auditory Comprehension in Patients With Primary Brain Tumor.” Human Brain Mapping 44, no. 4: 1603–1616. 10.1002/hbm.26161. 36515634 PMC9921237

[brb371243-bib-0057] Zhang, K. , T. Yang , Y. Xia , et al. 2024. “Molecular Determinants of Neurocognitive Deficits in Glioma: Based on 2021 WHO Classification.” Journal of Molecular Neuroscience 74, no. 1: 17. 10.1007/s12031-023-02173-4. 38315329 PMC10844410

[brb371243-bib-0058] Zhang, N. , M. Xia , T. Qiu , et al. 2018. “Reorganization of Cerebro‐Cerebellar Circuit in Patients With Left Hemispheric Gliomas Involving Language Network: A Combined Structural and Resting‐State Functional MRI Study.” Human Brain Mapping 39, no. 12: 4802–4819. 10.1002/hbm.24324. 30052314 PMC6866325

