# Supplementary material for: A Language Performance Model for Predicting Glioma Recurrence and Molecular Biomarkers: A Retrospective Cohort Study
Source: Brain Behav. 2026 Mar 2;16(3):e71243. doi: 10.1002/brb3.71243 (PMC12951359; doi:10.1002/brb3.71243)

**Supplementary Online Content**

**A Language Performance Model for Predicting Glioma Recurrence and Molecular Biomarkers: A Retrospective Cohort Study**

**Supplementary Table 1** Items and Scoring of Aphasia Battery of Chinese (ABC)

**Supplementary Table 2** Baseline Characteristics of Glioma Patients in IDH1/2 Status

**Supplementary Table 3** Baseline Characteristics of Glioma Patients in MGMT Status

**Supplementary Table** **4** Baseline Characteristics of Glioma Patients in 1p/19q Status

**Supplementary Table 5** Principal Component Analysis (PCA) on Language Tests Scores

**Supplementary Table 6** Predictive performance of models incorporating different numbers of principal components

**Supplementary Figure 1** AUC distribution and Quantile Quantile plots for LTC model based on Bootstrap

**Supplementary Figure 2** Time-dependent ROC analyses of the LTC model at different prediction horizons

**Supplementary Figure 3** Nomogram for Predicted 12-month Recurrence Probability in Glioma Patients

| **Supplementary Table 1** **Items and Scoring of Aphasia Battery of Chinese (ABC)** | | |
| --- | --- | --- |
| **Task** | **Description** | **Scores** |
| **Test 1. Spontaneous speech**  Audio recording: Patients should be encouraged to speak as much as possible, with a recording duration of at least 5–10 minutes. The examiner should avoid interrupting continuous speech. If no or only occasional grammatical function words appear within one minute, it is classified as “absent.” If fewer than half of the utterances within one minute contain grammatical function words, it is classified as “reduced.” | | |
| **Subtest 1. Conversational question** | The patient verbally responds to 6 personal questions (e.g., “How are you feeling now?”), scores assigned according to task difficulty. | 10 |
| **Subtest 2. Personal description** | 1.The patient describes pictures in the stimulus book. This item is scored 5 points.  2. The patient is asked to speak continuously and fluently about familiar topics such as their illness, work, or family. This item is scored 5 points. | 10 |
| **Test 2. Auditory verbal comprehension** | | |
| **Subtest 1. Yes/No questions** | This subtest includes 22 items.  Part 1 consists of 14 simple declarative statements about familiar facts (e.g., “You are a man.”). Each item is scored 2 points, and the patient answers “yes” or “no.”  Part 2 consists of 8 statements containing grammatical structures (e.g., “1kg flour is heavier than 2kg.”). Each item is scored 4 points, also requiring only “yes” or “no.” | 60 |
| **Subtest 2. Auditory recognition** | The patient is shown 5 pictures in the stimulus book. The patient must point to what the examiner says. A correct response within 5 seconds scores 2 points; a correct response after 5 seconds scores 1 point; no response or an incorrect response scores 0. | 90 |
| **Subtest 3. Sequential commands** | The patient performs actions according to spoken instructions. Complex commands must be delivered in full before the patient begins. There are 10 commands, with scores assigned according to task difficulty (e.g., “Raise your hand” scores 2 points; “Place the comb and pencil in a crossed position” scores 7 points). | 80 |
| **Test 3. Repetition**  The instruction is: “Please repeat after me. Say exactly what I say.” If the patient does not hear clearly, the entire word or sentence may be repeated. If speech articulation is impaired but the repetition is recognizable and correct, it is marked as correct. Phonemic paraphasias result in a deduction of half a point, with each word valued at one point. | | |
| **Subtest 1. Words repetition** | The patient must repeat words and phrases of increasing difficulty, with a total of 12 items, scores assigned according to task difficulty. | 24 |
| **Subtest 2. Sentences repetition** | The patient must repeat sentences of increasing difficulty, with a total of 9 items, scores assigned according to task difficulty. | 76 |
| **Test 4. Naming** | | |
| **Subtest 1. Object naming** | Patient is asked to name objects or pictures. If cannot name an item by sight, patient may be allowed to touch the object or body part to exclude the possibility of visual agnosia. | 60 |
| **Subtest 2. Responsive speech** | The patient must respond with the correct name. No additional explanation should be provided during questioning. Each of the 10 items is scored 2 points. | 20 |
| **Subtest 3. Word fluency** | The patient is asked to name as many vegetables as possible within one minute, and the total number produced is recorded. | 20 |
| **Test 5. Reading** | | |
| **Subtest 1. Reading words** | The patient must read out 10 words written on the card that presented to them. Each item is scored 1 point. | 10 |
| **Subtest 2. Spoken word-written word choice matching** | The examiner will say a word and the patient must point to correct word written on the card, then matches it. Each of 10 items is scored 2 points. | 10 |
| **Subtest 3. Written word stimulus-picture choice matching** | A card with words is shown to the patient. They are then asked to read it out and point to the picture that matches the word. Each of 20 items is scored 2 points. | 40 |
| **Subtest 4. Reading commands** | The patient is asked to read a command out loud and then do what it says. There are 5 items, scores assigned according to task difficulty. | 30 |
| **Subtest 5. Reading comprehension of sentences** | The patient is shown 5 sentences and asked to point to the best missing word from a list, to complete the sentence. There are 5 items, scores assigned according to task difficulty. | 30 |
| **Test 6. Writing** | | |
| **Subtest 1. Writing on request** | The patient must write down their name and address. | 10 |
| **Subtest 2. Copying a sentence** | The patient is shown a card with a sentence written on it. They are instructed to copy down this sentence. Each correct word is scored 2 points. | 10 |
| **Subtest 3. Series writing** | The patient is asked to write numbers correctly from 1 to 23. Each correctly written digit scores 1 point; omissions or reversals receive 0 points. | 20 |
| **Subtest 4. Writing to dictation** | Writing tasks include radicals, numbers, characters, words, and sentences. When dictating numbers, the patient may write either Arabic numerals or Chinese characters. Each of 40 items is scored 1 point. | 40 |
| **Subtest 5. Written output** | the examiner presents a picture and instructs the patient to “write what is shown in the picture.” The examiner may proceed item by item, pointing to the next image only after the patient completes the previous one. Each of 10 items is scored 2 points. | 20 |
| **Subtest 6. Spontaneous writing** | The patient must produce at least three complete sentences. The scoring criteria for short-text writing are as follows:  0 – No response or only lines.  1 – Scribbles resembling single characters with construction errors; no meaningful content.  2 – Presence of key words.  3 – Short phrases conveying some meaning.  4 – Occasional character-construction errors or grammatical inaccuracy, but includes complete sentences expressing information.  5 – Normal performance. | 5 |

| **Supplementary Table 2** **Baseline Characteristics of Glioma Patients in IDH1/2 Status** | | | | | |
| --- | --- | --- | --- | --- | --- |
| **Demographic and clinical data** | **Overall**  **(n=188)** | **IDH1/2 mutation (+/-)** | | | |
|  |  | **IDH1/2 (+)**  **(n=121)** | **IDH1/2 (-)**  **(n=67)** | **t/z/**$\boldsymbol{\chi}$**^2^** | ***p* value** |
| Age at diagnosis (years), mean±SD | 42.93±10.47 | 41.71±9.95 | 45.13±11.08 | -2.17 | 0.031* |
| Sex male/female | 110/78 | 68/53 | 42/25 | 0.75 | 0.387 |
| Education (years), mean±SD | 12.42±3.14 | 12.41±2.96 | 12.43±3.45 | -0.04 | 0.967 |
| WHO grade 2, n (%) | 114 (60.64) | 95 (78.51) | 19 (28.36) | 45.45 | 2.86×10^−11^** |
| WHO grade 3, n (%) | 30 (15.96) | 17 (14.05) | 13 (19.40) | 0.92 | 0.340 |
| WHO grade 4, n (%) | 44 (23.40) | 9 (7.44) | 35 (52.24) | 48.28 | 1.63×10^−11^** |
| Location (frontal lobe), n (%) | 86 (45.74) | 65 (53.72) | 21 (31.34) | 8.70 | 0.003* |
| Location (temporal lobe), n (%) | 27 (14.36) | 11 (9.09) | 16 (23.88) | 7.67 | 0.006* |
| Location (parietal lobe), n (%) | 15 (7.98) | 4 (3.31) | 11 (16.42) | 10.10 | 0.001* |
| Location (occipital lobe), n (%) | 4 (2.13) | 2 (1.65) | 2 (2.99) | 0.01 | 0.937 |
| Location (insular lobe), n (%) | 11 (5.85) | 8 (6.61) | 3 (4.48) | 0.07 | 0.785 |
| Recurrence (%) | 39 (20.74) | 9 (7.44) | 30 (44.78) | 36.57 | 1.07×10^−9^** |
| **Clinical symptoms, n (%)** |  |  |  |  |  |
| Headache | 58 (30.85) | 33 (27.27) | 25 (37.31) | 2.04 | 0.153 |
| Dizziness | 20 (10.64) | 13 (10.74) | 7 (10.45) | 0.00 | 0.950 |
| Nausea | 6 (3.19) | 4 (3.31) | 2 (2.99) | 0.00 | 1.000 |
| Vomiting | 8 (4.26) | 5 (4.13) | 3 (4.48) | 0.00 | 1.000 |
| Seizure | 20 (10.64) | 17 (14.05) | 3 (4.48) | 4.16 | 0.041* |
| Intracranial space-occupying  lesion or intracranial tumor | 6 (3.19) | 5 (4.13) | 1 (1.49) | 0.31 | 0.580 |
| Impaired consciousness | 57 (30.32) | 44 (36.36) | 13 (19.40) | 5.87 | 0.015* |
| Limb numbness | 18 (9.57) | 10 (8.26) | 8 (11.94) | 0.67 | 0.412 |
| Speech disorder | 13 (6.91) | 2 (1.65) | 11 (16.42) | 12.40 | 2.10×10^−4^** |
| Memory deterioration | 3 (1.60) | 1 (0.83) | 2 (2.99) | 0.27 | 0.601 |
| Visual impairment | 5 (2.66) | 1 (0.83) | 4 (5.97) | 2.64 | 0.104 |
| **Behavioral data, mean±SD** |  |  |  |  |  |
| KPS | 95.69±6.12 | 96.36±6.19 | 94.48±5.85 | 2.04 | 0.043* |
| MMSE | 26.27±3.48 | 26.99±2.80 | 24.97±4.16 | 3.55 | 3.52×10^−4^** |
| AQ | 92.45±7.16 | 94.15±4.69 | 89.37±9.50 | 3.86 | 1.18×10^−4^** |
| CQ | 92.97±8.66 | 95.18±4.79 | 88.97±12.07 | 4.04 | 6.14×10^−5^** |
| Spontaneous speech | 90.66±8.70 | 92.23±7.30 | 87.84±10.23 | 3.11 | 0.002* |
| Conversational question | 87.13±11.39 | 88.76±10.21 | 84.18±12.81 | 2.52 | 0.013* |
| Personal description | 94.20±8.46 | 95.70±7.05 | 91.49±10.04 | 3.04 | 0.003* |
| Repetition ^a^ | 96.36±7.22 | 97.74±4.60 | 93.85±9.96 | 3.03 | 0.003* |
| Sentences repetition | 95.20±9.48 | 97.02±6.07 | 91.91±13.06 | 3.03 | 0.003* |
| Naming | 90.83±9.10 | 92.82±5.74 | 87.24±12.43 | 3.47 | .0006** |
| Object naming | 95.71±6.79 | 97.23±3.66 | 92.96±9.71 | 3.47 | .0006** |
| Responsive speech | 94.26±10.13 | 95.54±7.52 | 91.94±13.40 | 2.03 | 0.046* |
| Word fluency | 73.01±23.14 | 77.07±20.01 | 65.67±26.54 | 3.06 | 0.003* |
| Auditory verbal comprehension | 93.76±8.52 | 95.91±5.10 | 89.88±11.61 | 4.04 | 6.14×10^−5^** |
| Yes/No questions | 93.91±7.27 | 95.02±6.06 | 91.91±8.76 | 2.58 | 0.011* |
| Auditory recognition | 96.81±5.03 | 97.89±3.26 | 94.87±6.82 | 3.42 | 0.0008** |
| Sequential commands | 90.20±17.28 | 94.22±10.99 | 82.93±23.31 | 3.74 | 1.77×10^−4^** |
| Reading | 94.55±10.46 | 96.92±5.21 | 90.27±15.23 | 3.46 | 1.11×10^−4^** |
| Reading words | 94.47±7.76 | 94.79±6.20 | 93.88±9.99 | .77 | 0.441 |
| Spoken word-written word choice matching | 96.97±11.56 | 99.34±3.35 | 92.69±18.14 | 2.97 | 0.004* |
| Written word stimulus-picture choice matching | 97.93±5.31 | 98.89±2.37 | 96.19±8.06 | 2.68 | 0.009* |
| Read out | 97.34±5.67 | 98.22±3.64 | 95.75±7.94 | 2.42 | 0.018* |
| Comprehension | 98.40±6.49 | 99.46±2.22 | 96.49±10.23 | 2.35 | 0.022* |
| Reading commands | 94.56±15.31 | 97.73±6.29 | 88.85±23.25 | 3.06 | 0.003* |
| Read out | 96.47±13.00 | 98.43±5.60 | 92.94±20.05 | 2.19 | 0.031* |
| Comprehension | 92.59±19.40 | 96.91±8.87 | 84.79±28.76 | 3.36 | 0.001* |
| Reading comprehension of sentences | 89.97±20.45 | 94.42±13.31 | 81.94±27.58 | 3.49 | 3.95×10^−4^** |
| Writing | 89.14±13.88 | 91.88±9.20 | 84.19±18.80 | 3.15 | 0.002* |
| Writing on request | 96.38±10.38 | 97.52±8.09 | 94.33±13.40 | 1.78 | 0.078 |
| Copying a sentence | 96.81±10.97 | 97.27±11.18 | 95.97±10.60 | .78 | 0.437 |
| Series writing | 99.49±7.52 | 100.00^a^ | 98.28±12.33 | 1.65 | 0.101 |
| Writing to dictation | 88.29±17.70 | 91.90±10.89 | 81.76±24.59 | 3.21 | 0.002* |
| Written output | 77.66±28.09 | 81.98±22.43 | 69.85±35.00 | 2.56 | 0.012* |
| Spontaneous writing | 72.98±35.19 | 78.68±31.38 | 62.69±39.37 | 2.86 | 0.005* |
| Abbreviations: KPS=Karnofsky performance status; MMSE=Mini-Mental State Examination; AQ=Aphasia Quotient; CQ=Cortical Quotient; IDH1/2=isocitrate dehydrogenase 1/2.  Language scores have been normalized into percentages based on their full marks, which refer to mini-mental state examination and Aphasia Battery of Chinese (ABC), which adapted from the Western Aphasia Battery (WAB). Behavior data were obtained for the day before the surgery, all patients underwent assessment which performed by an experienced neuropsychologist.  ^a^ All patients have achieved perfect scores in words repetition, which was not shown in the table; all patients with IDH1 (+) have achieved perfect scores in series writing.  Continuous data are shown as mean±SD, with statistical significance based on two sample t test. Categorical data differences (No. and percentages) are represented with statistical significance based on chi-squared test ($\chi$^2^ & *p*) and Fisher exact test (z & *p*).  *p<*0.05 was considered statistically significant and marked with an asterisk (*), *: *p*<0.05, **: *p*<0.001. | | | | | |

| **Supplementary Table 3** **Baseline Characteristics of Glioma Patients in MGMT Status** | | | | | |
| --- | --- | --- | --- | --- | --- |
| **Demographic and clinical data** | **Overall**  **(n=60)** | **MGMT promoter methylation (+/-)**^a^ | | | |
|  |  | **MGMT (+)**  **(n=9)** | **MGMT (-)**  **(n=51)** | **t/z/**$\boldsymbol{\chi}$**^2^** | ***p* value** |
| Age at diagnosis (years), mean±SD | 45.93±9.87 | 46.00±11.50 | 45.92±9.69 | 0.02 | 0.983 |
| Sex male/female | 30/30 | 5/4 | 25/26 | 0.00 | 1.000 |
| Education (years), mean±SD | 12.78±3.56 | 12.22±4.55 | 12.88±3.40 | -0.51 | 0.612 |
| WHO grade 2, n (%) | 39 (65.00) | 7 (77.78) | 32 (62.75) | 0.24 | 0.620 |
| WHO grade 3, n (%) | 10 (16.67) | 1 (11.11) | 9 (17.65) | 0.00 | 1.000 |
| WHO grade 4, n (%) | 11 (18.33) | 1 (11.11) | 10 (19.61) | 0.02 | 0.889 |
| Location (frontal lobe), n (%) | 30 (50.00) | 7 (77.78) | 23 (45.10) | 2.09 | 0.148 |
| Location (temporal lobe), n (%) | 7 (11.67) | 1 (11.11) | 6 (11.76) | 0.00 | 1.000 |
| Location (parietal lobe), n (%) | 8 (13.33) | 1 (11.11) | 7 (13.73) | 0.00 | 1.000 |
| Location (occipital lobe), n (%) | 4 (6.67) | 0 (0.00) | 4 (7.84) | 0.00 | 1.000 |
| Location (insular lobe), n (%) | 2 (3.33) | 2 (22.22) | 0 (0.00) | 7.82 | 0.020* |
| Recurrence (%) | 14 (23.33) | 3 (33.33) | 11 (21.57) | 0.12 | 0.732 |
| **Clinical symptoms, n (%)** |  |  |  |  |  |
| Headache | 19 (31.67) | 1 (11.11) | 18 (35.29) | 1.10 | 0.294 |
| Dizziness | 6 (10.00) | 1 (11.11) | 5 (9.80) | 0.00 | 1.000 |
| Nausea | 3 (5.00) | 1 (11.11) | 2 (3.92) | 1.87 | 0.391 |
| Vomiting | 4 (6.67) | 1 (11.11) | 3 (5.88) | 1.44 | 0.488 |
| Seizure | 5 (8.33) | 2 (22.22) | 3 (5.88) | 3.69 | 0.158 |
| Intracranial space-occupying  lesion or intracranial tumor | 3 (5.00) | 0 (0.00) | 3 (5.88) | 0.00 | 1.000 |
| Impaired consciousness | 21 (35.00) | 5 (55.56) | 16 (31.37) | 1.05 | 0.306 |
| Limb numbness | 7 (11.67) | 0 (0.00) | 7 (13.73) | 0.38 | 0.536 |
| Speech disorder | 2 (3.33) | 1 (11.11) | 1 (1.96) | 2.54 | 0.280 |
| Memory deterioration | 1 (1.67) | 1 (11.11) | 0 (0.00) | 3.79 | 0.150 |
| Visual impairment | 2 (3.33) | 0 (0.00) | 2 (3.92) | 0.00 | 1.000 |
| **Behavioral data, mean±SD** |  |  |  |  |  |
| KPS | 95.67±6.21 | 92.22±9.72 | 96.27±5.28 | -1.84 | 0.071 |
| MMSE | 26.05±3.62 | 25.00±3.24 | 26.24±3.68 | -0.94 | 0.350 |
| AQ | 92.27±6.17 | 89.22±7.51 | 92.80±5.83 | -1.63 | 0.109 |
| CQ | 93.25±7.21 | 92.22±5.04 | 93.43±7.56 | -0.46 | 0.647 |
| Spontaneous speech | 90.08±8.10 | 86.11±9.61 | 90.78±7.71 | -1.62 | 0.111 |
| Conversational question | 86.67±10.20 | 83.33±11.18 | 87.25±10.02 | -1.07 | 0.291 |
| Personal description | 93.50±8.80 | 88.89±12.69 | 94.31±7.81 | -1.73 | 0.088 |
| Repetition ^a^ | 96.15±6.56 | 92.67±9.89 | 96.76±5.70 | -1.76 | 0.084 |
| Sentences repetition | 94.93±8.65 | 90.33±12.92 | 95.75±7.55 | -1.76 | 0.083 |
| Naming | 91.00±7.54 | 89.22±8.20 | 91.31±7.46 | -0.76 | 0.447 |
| Object naming | 96.43±5.61 | 96.11±5.60 | 96.49±5.67 | -0.19 | 0.854 |
| Responsive speech | 95.00±9.48 | 92.22±15.63 | 95.49±8.08 | -0.95 | 0.345 |
| Word fluency | 71.00±22.15 | 65.56±18.78 | 71.96±22.72 | -0.80 | 0.428 |
| Auditory verbal comprehension | 94.12±7.21 | 92.44±5.46 | 94.41±7.48 | -0.75 | 0.455 |
| Yes/No questions | 93.38±7.31 | 91.22±6.02 | 93.76±7.50 | -0.96 | 0.340 |
| Auditory recognition | 97.45±4.26 | 97.78±2.86 | 97.39±4.48 | 0.25 | 0.805 |
| Sequential commands | 90.90±14.90 | 87.56±12.00 | 91.49±15.38 | -0.73 | 0.470 |
| Reading | 94.38±10.15 | 94.56±8.53 | 94.35±10.49 | 0.05 | 0.957 |
| Reading words | 94.00±8.07 | 93.33±7.07 | 94.12±8.29 | -0.27 | 0.791 |
| Spoken word-written word choice matching | 97.17±9.93 | 100.00^a^ | 96.67±10.71 | 0.93 | 0.358 |
| Written word stimulus-picture choice matching | 98.57±2.81 | 99.44±1.67 | 97.84±4.03 | 1.17 | 0.248 |
| Read out | 98.08±3.81 | 100.00^a^ | 98.73±3.44 | 1.10 | 0.274 |
| Comprehension | 98.92±3.20 | 96.22±9.01 | 96.73±15.17 | -0.10 | 0.924 |
| Reading commands | 94.98±14.43 | 96.33±7.48 | 92.65±18.96 | 0.57 | 0.569 |
| Read out | 96.65±14.35 | 96.33±11.00 | 87.88±22.15 | 1.11 | 0.270 |
| Comprehension | 93.20±17.72 | 100.00^a^ | 96.67±10.71 | 0.93 | 0.358 |
| Reading comprehension of sentences | 89.15±21.01 | 99.44±1.67 | 97.84±4.03 | 1.17 | 0.248 |
| Writing ^a^ | 90.18±12.01 | 90.22±11.45 | 90.18±12.22 | 0.01 | 0.992 |
| Writing on request | 99.00±5.43 | 100.00^a^ | 98.82±5.88 | 0.60 | 0.554 |
| Copying a sentence | 97.00±14.99 | 100.00^a^ | 96.47±16.23 | 0.65 | 0.520 |
| Writing to dictation | 88.98±17.52 | 89.22±17.72 | 88.94±17.66 | 0.04 | 0.965 |
| Written output | 79.17±24.58 | 80.00±22.91 | 79.02±25.08 | 0.11 | 0.913 |
| Spontaneous writing | 74.67±35.10 | 62.22±41.77 | 76.86±33.79 | -1.16 | 0.252 |
| Abbreviations: KPS=Karnofsky performance status; MMSE=Mini-Mental State Examination; AQ=Aphasia Quotient; CQ=Cortical Quotient; MGMT=O-6-methylguanine-DNA methyltransferase promoter methylation.  Language scores have been normalized into percentages based on their full marks, which refer to mini-mental state examination and Aphasia Battery of Chinese (ABC), which adapted from the Western Aphasia Battery (WAB). Behavior data were obtained for the day before the surgery, all patients underwent assessment which performed by an experienced neuropsychologist.  ^a^ All patients have achieved perfect scores in Words repetition and Series writing, which were not shown in the table; all patients with MGMT promoter methylation have achieved perfect scores in Spoken word-written word choice matching, Read out (Written word stimulus-picture choice matching), Comprehension (Reading commands), Writing on request, and Copying a sentence.  Continuous data are shown as mean±SD, with statistical significance based on two sample t test. Categorical data differences (No. and percentages) are represented with statistical significance based on chi-squared test ($\chi$^2^ & *p*) and Fisher exact test (z & *p*).  *p<*0.05 was considered statistically significant and marked with an asterisk (*), *: *p*<0.05, **: *p*<0.001. | | | | | |

| **Supplementary Table 4** **Baseline Characteristics of Glioma Patients in 1p/19q Status** | | | | | |
| --- | --- | --- | --- | --- | --- |
| **Demographic and clinical data** | **Overall**  **(n=70)** | **1p/19q (noncodeletion/ codeletion)**^a^ | | | |
|  |  | **1p/19q-noncodeletion**  **(n=48)** | **1p1/9q-codeletion^a^**  **(n=22)** | **t/z/**$\boldsymbol{\chi}$**^2^** | ***p* value** |
| Age at diagnosis (years), mean±SD | 43.43±9.79 | 44.54±9.18 | 41.00±10.82 | 1.42 | 0.161 |
| Sex male/female | 40/30 | 30/18 | 10/12 | 1.79 | 0.181 |
| Education (years), mean±SD | 13.36±3.53 | 13.90±3.37 | 12.18±3.65 | 1.92 | 0.058 |
| WHO grade 2, n (%) | 44 (62.86) | 25 (52.08) | 19 (86.36) | 7.59 | 0.006* |
| WHO grade 3, n (%) | 13 (18.57) | 12 (25.00) | 1 (4.55) | 2.93 | 0.087 |
| WHO grade 4, n (%) | 13 (18.57) | 11 (22.92) | 2 (9.09) | 1.10 | 0.294 |
| Location (frontal lobe), n (%) | 31 (44.29) | 19 (39.58) | 12 (54.55) | 1.37 | 0.242 |
| Location (temporal lobe), n (%) | 7 (10.00) | 5 (10.42) | 2 (9.09) | 0.00 | 1.000 |
| Location (parietal lobe), n (%) | 8 (11.43) | 5 (10.42) | 3 (13.64) | 0.00 | 1.000 |
| Location (occipital lobe), n (%) | 2 (2.86) | 2 (4.17) | 0 (0.00) | 0.00 | 1.000 |
| Location (insular lobe), n (%) | 5 (7.14) | 4 (8.33) | 1 (4.55) | 0.01 | 0.943 |
| Recurrence (%) | 21 (30.00) | 17 (35.42) | 4 (18.18) | 2.13 | 0.144 |
| **Clinical symptoms**^a^**, n (%)** |  |  |  |  |  |
| Headache | 25 (35.71) | 18 (37.50) | 7 (31.82) | 0.21 | 0.645 |
| Dizziness | 7 (10.00) | 6 (12.50) | 1 (4.55) | 0.36 | 0.548 |
| Nausea | 3 (4.29) | 2 (4.17) | 1 (4.55) | 0.00 | 1.000 |
| Vomiting | 4 (5.71) | 3 (6.25) | 1 (4.55) | 0.00 | 1.000 |
| Seizure | 6 (8.57) | 4 (8.33) | 2 (9.09) | 0.00 | 1.000 |
| Intracranial space-occupying  lesion or intracranial tumor | 4 (5.71) | 3 (6.25) | 1 (4.55) | 0.00 | 1.000 |
| Impaired consciousness | 23 (32.86) | 14 (29.17) | 9 (40.91) | 0.94 | 0.332 |
| Limb numbness | 10 (14.29) | 5 (10.42) | 5 (22.73) | 1.00 | 0.318 |
| Speech disorder | 3 (4.29) | 3 (6.25) | 0 (0.00) | 1.20 | 0.547 |
| **Behavioral data** |  |  |  |  |  |
| KPS | 96.00±6.00 | 95.83±5.39 | 96.36±7.27 | -0.34 | 0.734 |
| MMSE | 26.69±3.22 | 26.81±3.07 | 26.41±3.59 | 0.48 | 0.630 |
| AQ | 92.97±6.14 | 93.12±5.75 | 92.64±7.05 | 0.31 | 0.760 |
| CQ | 94.09±7.02 | 94.48±5.54 | 93.23±9.60 | 0.69 | 0.493 |
| Spontaneous speech | 90.57±7.54 | 90.73±7.72 | 90.23±7.32 | 0.26 | 0.798 |
| Conversational question | 86.57±10.48 | 86.88±10.55 | 85.91±10.54 | 0.36 | 0.723 |
| Personal description | 94.57±7.36 | 94.58±7.13 | 94.55±8.00 | 0.02 | 0.984 |
| Repetition^b^ | 96.46±7.43 | 95.81±8.17 | 97.86±5.36 | -1.07 | 0.287 |
| Sentences repetition | 95.31±9.81 | 94.46±10.77 | 97.18±7.14 | -1.08 | 0.284 |
| Naming | 92.19±8.17 | 92.81±7.25 | 90.82±9.94 | 0.95 | 0.347 |
| Object naming | 96.47±6.29 | 96.92±5.68 | 95.50±7.51 | 0.87 | 0.386 |
| Responsive speech | 95.71±8.44 | 96.46±6.35 | 94.09±11.82 | 1.09 | 0.279 |
| Word fluency | 76.07±21.73 | 77.08±20.98 | 73.86±23.65 | 0.57 | 0.569 |
| Auditory verbal comprehension | 94.96±7.08 | 95.42±5.03 | 93.95±10.31 | 0.80 | 0.426 |
| Yes/No questions | 94.34±6.92 | 94.62±6.61 | 93.73±7.69 | 0.50 | 0.618 |
| Auditory recognition | 97.67±4.59 | 97.85±3.80 | 97.27±6.06 | 0.49 | 0.626 |
| Sequential commands | 92.34±14.55 | 93.27±10.02 | 90.32±21.56 | 0.79 | 0.435 |
| Reading | 95.59±6.55 | 95.94±6.03 | 94.82±7.66 | 0.66 | 0.511 |
| Reading words | 95.29±6.53 | 98.75±4.89 | 98.64±4.68 | 0.09 | 0.927 |
| Spoken word-written word choice matching | 98.71±4.79 | 98.23±3.93 | 97.50±4.56 | 0.69 | 0.496 |
| Written word stimulus-picture choice matching | 98.40±3.47 | 98.96±4.61 | 98.18±3.95 | 0.68 | 0.497 |
| Read out | 98.00±4.12 | 98.46±6.50 | 98.45±4.61 | 0.00 | 0.998 |
| Comprehension | 98.71±4.40 | 97.08±8.13 | 91.50±22.21 | 1.54 | 0.128 |
| Reading commands | 96.91±9.04 | 89.92±17.42 | 88.59±17.57 | 0.29 | 0.769 |
| Read out | 98.46±5.93 | 98.75±4.89 | 98.64±4.68 | 0.09 | 0.927 |
| Comprehension | 95.33±14.21 | 98.23±3.93 | 97.50±4.56 | 0.69 | 0.496 |
| Reading comprehension of sentences | 89.50±17.35 | 98.96±4.61 | 98.18±3.95 | 0.68 | 0.497 |
| Writing | 90.93±12.22 | 91.77±9.55 | 89.09±16.78 | 0.85 | 0.398 |
| Writing on request | 98.29±7.01 | 98.33±9.07 | 99.55±2.13 | -0.62 | 0.539 |
| Copying a sentence | 98.71±7.60 | 100.00^b^ | 94.77±21.41 | 1.71 | 0.092 |
| Series writing | 98.36±12.06 | 100.00^b^ | 94.77±21.41 | 1.71 | 0.092 |
| Writing to dictation | 91.01±14.37 | 92.02±11.43 | 88.82±19.47 | 0.86 | 0.391 |
| Written output | 80.79±25.13 | 81.35±23.26 | 79.55±29.35 | 0.28 | 0.782 |
| Spontaneous writing | 73.43±33.83 | 72.92±33.39 | 74.55±35.55 | -0.19 | 0.853 |
| Abbreviations: KPS=Karnofsky performance status; MMSE=Mini-Mental State Examination; AQ=Aphasia Quotient; CQ=Cortical Quotient; 1p/19q-codeletion=complete deletion of complete deletion of both the short arm of chromosome 1 (1p) and the long arm of chromosome 19 (19q).  Language scores have been normalized into percentages based on their full marks, which refer to mini-mental state examination and Aphasia Battery of Chinese (ABC), which adapted from the Western Aphasia Battery (WAB). Behavior data were obtained for the day before the surgery, all patients underwent assessment which performed by an experienced neuropsychologist.  ^a^ There were no patients with memory deterioration or visual impairment in 1p/19q status.  ^b^ All patients have achieved perfect scores in words repetition, which were not shown in the table; all patients with 1p/19q-noncodeletion have achieved perfect scores in copying a sentence and series writing.  Continuous data are shown as mean±SD, with statistical significance based on two sample t test. Categorical data differences (No. and percentages) are represented with statistical significance based on chi-squared test ($\chi$^2^ & *p*) and Fisher exact test (z & *p*).  *p<*0.05 was considered statistically significant and marked with an asterisk (*), *: *p*<0.05, **: *p*<0.001. | | | | | |

| **Supplementary Table 5** **Principal Component Analysis (PCA) on Language Tests Scores** | | | | | |
| --- | --- | --- | --- | --- | --- |
| **Variables** | **Auditory verbal comprehension & Writing^†^** | **Repetition^†^** | **Series writing & Sequential commands^†^** | **Naming^†^** | **Personal description & Series writing^†^** |
| Sentences repetition | 0.243 | 0.577 | 0.121 | -0.060 | 0.045 |
| Repetition | 0.244 | 0.577 | 0.118 | -0.056 | 0.048 |
| Auditory verbal comprehension | 0.326 | 0.020 | 0.123 | -0.257 | -0.212 |
| Naming | 0.308 | -0.051 | -0.023 | 0.426 | -0.247 |
| Object naming | 0.289 | -0.072 | 0.024 | 0.550 | -0.292 |
| Series writing | 0.131 | -0.327 | 0.803 | 0.020 | 0.386 |
| Personal description | 0.238 | 0.191 | -0.186 | 0.320 | 0.666 |
| Writing | 0.312 | -0.234 | -0.137 | 0.006 | 0.178 |
| Reading comprehension of sentences | 0.248 | -0.167 | -0.454 | -0.275 | 0.197 |
| Reading commands (comprehension) | 0.298 | -0.170 | -0.041 | -0.270 | -0.104 |
| Auditory recognition | 0.300 | -0.096 | 0.019 | 0.134 | -0.259 |
| Writing to dictation | 0.309 | -0.243 | -0.151 | -0.091 | 0.178 |
| Sequential commands | 0.300 | 0.052 | 0.162 | -0.407 | -0.177 |
| % Variance | 0.612 | 0.109 | 0.071 | 0.046 | 0.045 |
| % Cumulative variance | 0.612 | 0.721 | 0.792 | 0.838 | 0.883 |
| ^†^ The top five components, which collectively contributed to 88.3% of the variance, were selected to represent the language scores in the multivariable Cox model. The components were named according to the two indices with the largest coefficients. | | | | | |

| **Supplementary Table 6 Predictive performance of models incorporating different numbers of principal components** | | |
| --- | --- | --- |
| **Number of principal components (PCs) included** | **AUC (95% CI)^b^** | ***p* value** |
| 1 | 0.756 (0.616-0.831) | 1.00×10^−4^** |
| 2 | 0.778 (0.638-0.846) | 2.42×10^−5^** |
| 3 | 0.780 (0.633-0.844) | 4.83×10^−5^** |
| 4 | 0.779 (0.633-0.850) | 8.19×10^−5^** |
| 5^a^ | 0.834 (0.681-0.871) | 0.020* |
| ^a^ The model including five principal components corresponds to the final Language Tests Combinations (LTC) model.  ^b^ AUCs and corresponding 95% CI were estimated by resampling the data 3,000 times using the Bootstrap method.  *p<*0.05 was considered statistically significant and marked with an asterisk (*), *: *p*<0.05, **: *p*<0.001. | | |

**Supplementary Figure 1 AUC distribution and Quantile Quantile plots for LTC model based on Bootstrap**

The AUC distribution and Quantile Quantile plots were generated for the LTC model based on internal validation with 3000 resamples. The mean AUC for multiple resampling was 0.824, with a 95% confidence interval of (0.805, 0.863). Quantile–quantile plot of predicted versus observed probabilities for the LTC model based on 3,000 bootstrap resamples; points closely align with the diagonal line, indicating good calibration.


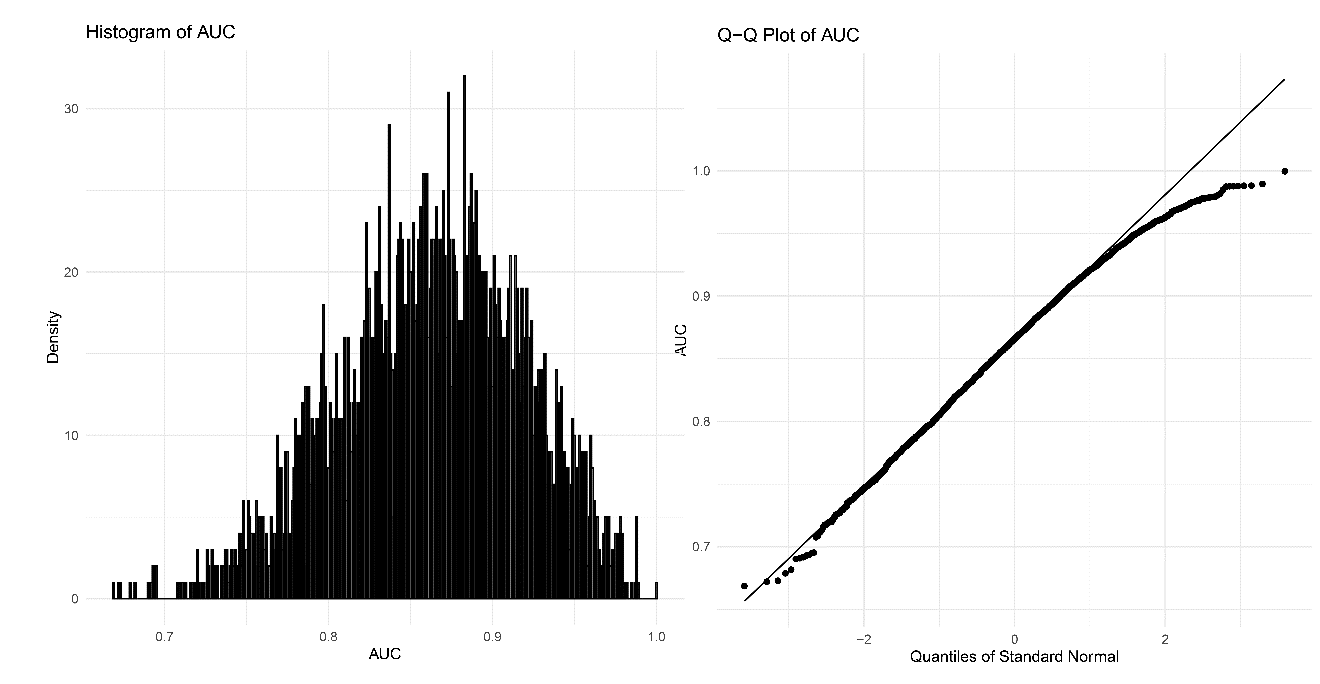


**Supplementary Figure 2 Time-dependent ROC analyses of the LTC model at different prediction horizons**

Time-dependent receiver operating characteristic (ROC) curves evaluating the discriminative performance of the LTC model for glioma recurrence at 12 months, 24 months, and over the entire follow-up period (8 years). Model performance is summarized by the area under the curve (AUC) with corresponding 95% confidence intervals, demonstrating good short-term predictive ability and sustained, stable long-term predictive performance.


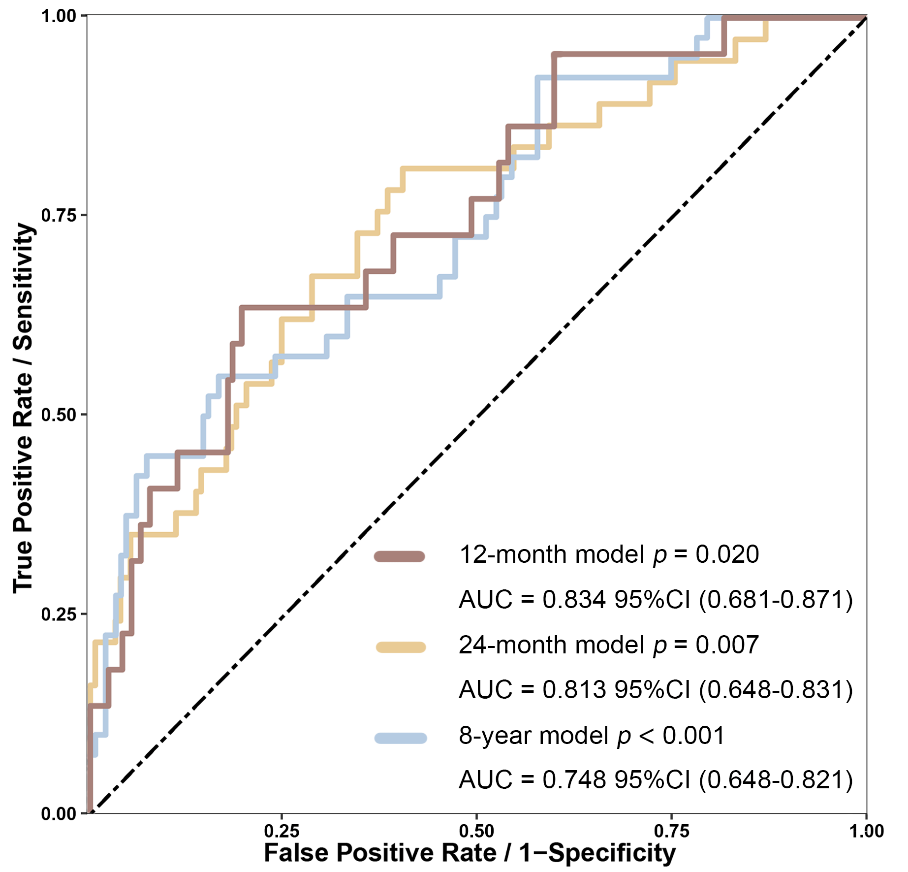


**Supplementary Figure 3 Nomogram for Predicted 12-month Recurrence Probability in Glioma Patients**

Sex (1=male, 0=female); age at diagnosis: age at the time of diagnosis; location (frontal lobe) (1=yes, 0=no); location (temporal lobe) (1=yes, 0=no); location (parietal lobe) (1=yes, 0=no). For showing the 12-month recurrence probability range, the total points range is shown as 220-230 points. Taking an example, a man, diagnosed with glioma at the age of 48 with an education level of 14 years, presented with a solitary lesion located in the frontal lobe. His KPS score is 90, and scored 2 points on Auditory recognition & Writing, scored 7 points on Naming, scored 1 point on Series writing & Sequential commands, and scored 1 point on Repetition, and scored 1 on Personal description & Series writing. respectively. Drawing a straight line from each score position on the respective axes, we obtain corresponding points. Summing these points yields a total of 221 points. Drawing a straight line downwards from 221 on the total points axis, it intersects with the12-month glioma recurrence probability axis, revealing the patient's 12-month glioma recurrence probability to be 0.90.


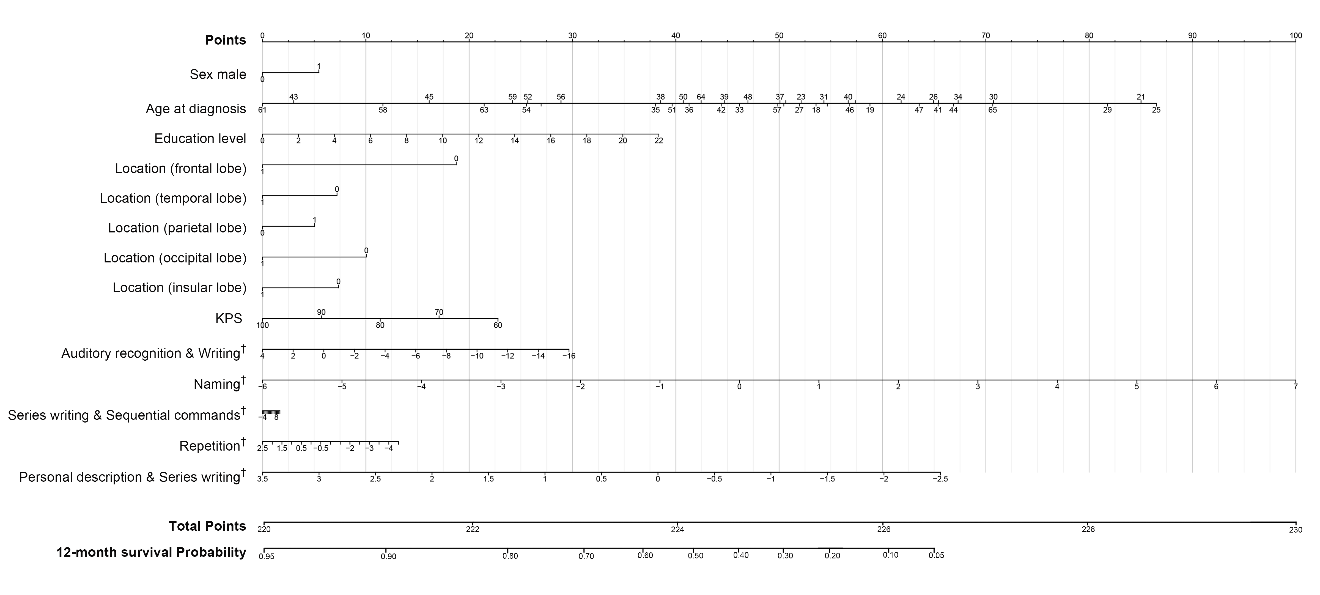

Supplement: Supplementary file 1 — Table S1 Items and scoring of the Aphasia Battery of Chinese (ABC). Table S2 Baseline characteristics of glioma patients in idh1/2 status. Table S3 Baseline characteristics of glioma patients in MGMT status. Table S4 Baseline characteristics of glioma patients in 1p/19q status. Table S5 Principal component analysis (PCA) on language test scores. Table S6 Predictive performance of models incorporating different numbers of principal components. Figure S1 AUC distribution and quantile plots for the LTC model based on bootstrap. Figure S2 Time‐dependent ROC analyses of the LTC model at different prediction horizons. Figure S3 Nomogram for predicted 12‐month recurrence probability in glioma patients. [file BRB3-16-e71243-s001.docx]
